# The autoimmune regulator (AIRE) is a target of the E3-Ubiquitin ligase Seven-in-absentia homolog 1 (SIAH1).

**DOI:** 10.1007/s10495-026-02398-9

**Published:** 2026-07-14

**Authors:** Adrián Tirado-Herranz, Alba Pastor-Moreno, María Area-Navarro, Jacqueline Noboa-Velástegui, Oscar Conchillo, José Ramón Palacio, Xavier Daura, Iñaki Alvarez

**Affiliations:** 1 Department of Cell Biology, Physiology, and Immunology, Universitat Autònoma de Barcelona, 08193 Bellaterra, Spain; 2 Institute of Biotechnology and Biomedicine, Universitat Autònoma de Barcelona, 08193 Bellaterra, Spain; 3 Department of Molecular Biology and Genomics, Centro Universitario de Ciencias de la Salud, Universidad de Guadalajara, Guadalajara, México; 4 Catalan Institution for Research and Advanced Studies (ICREA), Barcelona, Spain; 5 CIBER de Bioingeniería, Biomateriales y Nanomedicina, Instituto de Salud Carlos III, 08193 Bellaterra, Spain

**Keywords:** Thymus, Autoimmune regulator (AIRE), E3-ubiquitin ligase SIAH, Antigen processing, Apoptosis, Central tolerance

## Abstract

The autoimmune regulator (AIRE) is expressed in medullary thymic epithelial cells (mTECs) and is crucial for generating an immunocompetent T cell repertoire during central tolerance. Loss of AIRE function causes autoimmune polyglandular syndrome type 1 (APS-1), also known as autoimmune polyendocrinopathy-candidiasis-ectodermal dystrophy (APECED). Intracellular proteins are regulated by ubiquitination pathways. E3 ubiquitin ligases are specific proteins that confer selectivity to polyubiquitination, tagging proteins for proteasomal degradation while also performing other functions. In mTECs, peptides generated via the class I antigen-processing pathway are presented by HLA-I molecules to CD8 thymocytes to eliminate self-reactive T-cell precursors. AIRE induces promiscuous gene expression in mTECs, but little is known about the regulatory mechanisms of AIRE. Previously, we and others demonstrated that AIRE is an apoptosis inductor. Here, we demonstrate that AIRE increases the intracellular levels of SIAH-interacting protein (SIP), an adaptor protein of the SIAH E3 ubiquitin ligase family. We also show that AIRE interacts with SIAH1 in the human thymus. While AIRE contains two putative SIAH-interacting motifs, it interacts with SIAH proteins only through the sequence spanning residues 119–125. AlphaFold modeling indicated that the interaction between AIRE and SIAH1 is highly analogous to that observed between SIP and SIAH1, suggesting that both proteins compete for SIAH1 binding. The CARD domain and the SIAH-interacting motif is required to AIRE-mediated apoptosis. AIRE co-localized with SIAH proteins in the cytoplasm of HEK-293 cells. Finally, SIAH1 ubiquitinated AIRE, targeting it for proteasomal degradation. Collectively, these findings reveal a novel pathway for the degradation and regulation of AIRE.

**Supplementary Information:**

The online version contains supplementary material available at 10.1007/s10495-026-02398-9.

## Introduction

T cell progenitors exit the bone marrow and migrate to the thymus, where they undergo maturation, developing into naïve T cells. During this maturation process, potentially self-reactive thymocytes are eliminated in the thymic medulla through a mechanism known as negative selection. This involves the interaction of single positive thymocytes with self-peptide-MHC complexes (pMHC) presented by medullary thymic epithelial cells (mTECs) and medullary dendritic cells (mDCs) [1]. Thus, the final goal of thymic selection is the generation of an immunocompetent T cell repertoire that does not react against self-molecules and presents variability enough to react against many pathogens or aberrant cells. Ideally, all the self-proteome that T cells might encounter in the periphery should be expressed in the thymic medulla to efficiently eliminate self-reactive thymocytes.

The autoimmune regulator (AIRE) is predominantly expressed in mTECs, although it is also found in other extrathymic lymphoid tissues, including secondary lymphoid organs [2–4], the testis [5], and stem cells [6]. Loss of function of AIRE causes a disease called autoimmune polyglandular syndrome type 1 (APS-1), also denominated as autoimmune polyendocrinopathy candidiasis ectodermal dystrophy (APECED) (OMIM entry # 240300) [7, 8], a rare disease affecting children and teenagers described in 1963 [9]. APECED patients can develop most of the known autoimmune disorders. Diagnosis is established by the presence of at least two of the following symptoms: (i) candida infection, (ii) autoimmune hypoparathyroidism, and (iii) autoimmune Addison’s disease. APECED presents a higher prevalence among some populations such as Iranian Jews (1/9000) [10], Sardinians (1/14400) [11], and Finns (1/25000) [12].

AIRE controls the expression of many tissue-restricted antigens (TRAs) by mTECs, although the mechanism is not fully understood [7, 12, 13]. It has been reported that AIRE unleashes stalled RNA II polymerase [14], facilitates transcription and pre-mRNA processing [15], and interacts with specific unmethylated histones associated with inactive chromatin [16]. In addition, AIRE has been described as playing a role during differentiation and maturation of mTECs [17, 18].

AIRE-expressing cells exhibit increased apoptosis [19], suggesting the possibility that mDCs also present peptides to thymocytes derived from TRAs through cross-presentation [20]. In a previous analysis by our group, an increase of apoptosis was detected in AIRE-transfected HT93 cells (epithelial cells derived from human thyroid) [21]. One of the proteins detected as more abundant in AIRE^+^ cells was calcyclin-binding protein, or SIAH-interacting protein (SIP), an adaptor protein of the RING domain E3-ubiquitin ligase seven-in-absentia homolog (SIAH) family [22]. There are two functional proteins of this family, SIAH1 and SIAH2, which share some ligands but have also specific target proteins. E3 ubiquitin ligases are the members that confer specificity to the process of protein polyubiquitination, marking proteins for degradation in the proteasome. Furthermore, SIAH proteins have been involved in many cellular processes, such as DNA damage [23], hypoxia [24], RAS signaling [25], transcription [26], and p38 and JNK/NF-κB pathways [27, 28]. SIAH1 and SIAH2 are expressed in several thymic cell types, including AIRE^+^ mTECs [29].

Proteins that interact with SIAH members contain the consensus motif PxAxVxP, where x represents any amino acid. SIP, for instance, harbors the motif PAAVVAP at residues 60–66. The structural interaction between SIP and SIAH1 has been characterized [30, 31].

Here, using the HEK-293 cell model, we observed that AIRE induced an elevation of SIP levels, validating the findings in HT93 cells. Additionally, we investigated the direct interaction of AIRE with SIP and SIAH proteins. AIRE did not exhibit interaction with SIP, but it did with SIAH1 and SIAH2. The SIAH-interacting motif present in AIRE was defined. AlphaFold interaction modeling indicated that the interaction between AIRE and SIAH1 is highly analogous to that observed between SIAH1 and SIP. Moreover, SIAH1 ubiquitinated AIRE, tagging it to proteasomal degradation. This interaction was also detected in the human thymus, indicating that it occurs in vivo. Our data demonstrate that AIRE is a novel SIAH1-interacting protein, identifying a novel putative pathway of AIRE regulation.

## Materials and methods

### Antibodies

The following antibodies were used:

**Table:** 

Specificity	Isotype	Company	Cat. number	RRID
Anti-SIP (Clone D-8)	Mouse monoclonal IgG2a	Santa Cruz Biotechnology	sc-166,195	AB_2009821
Anti-Flag (Clone M2)	Mouse monoclonal IgG1	Sigma-Aldrich	F1804	AB_262044
Anti-6x His Tag (Clone HIS.H8)	Mouse monoclonal antibody	Invitrogen	MA1-21315	AB_557403
Anti-Vinculin (Clone 7F9)	Mouse monoclonal IgG antibody	Invitrogen	MA5-11690	AB_10976821
Anti-β-catenin (Clone 5H10)	Mouse monoclonal antibody	Invitrogen	13-8400	AB_2533039
Anti-β actin (Clone C4)	Mouse monoclonal antibody	Santa Cruz Biotechnology	sc-47,778	AB_626632
Anti-AIRE-1	Rabbit polyclonal IgG antibody	Invitrogen	PA5-78747	AB_2745863
Anti-Ubiquitin	Rabbit polyclonal antiserum	Sigma-Aldrich	U5379	AB_477667
Anti-SIAH1	rabbit polyclonal antibody	Antibodies.com	A89218	AB_2892180
ECL anti-Mouse IgG	Horseradish Peroxidase-Linked whole antibody from sheep	GE Healthcare - Cytiva	NA931	AB_772210
ECL anti-Rabbit IgG	Horseradish Peroxidase-Linked whole antibody from donkey	GE Healthcare - Cytiva	NA934	AB_772206
Alexa Fluor 488 anti-mouse IgG	Goat polyclonal antibody IgG (H + L)	Invitrogen (Thermo Fisher)	A-11,001	AB_2534069
Alexa Fluor 488 anti-Rabbit IgG	Goat polyclonal antibody IgG (H + L)	Invitrogen (Thermo Fisher)	A-11,008	AB_143165
Alexa Fluor 568 anti-mouse IgG	Goat polyclonal antibody IgG (H + L)	Invitrogen (Thermo Fisher)	A-11,004	AB_2534072

### DNA constructs

The pcDNA3.1-Flag-SIAH1 and pcDNA3.1-Flag-SIAH2 constructs were kindly gifted by Dr. Thilo Hagen (National University of Singapore (NUS) - Singapore).

The human *AIRE* gene and the constructs containing different regions of human *AIRE* were cloned in the pcDNA3.1-MycHis expression vector. The following constructs were used: AIRE-MycHis, AIRE-(1-106)-MycHis, AIRE-(107–545)-MycHis, AIRE-(1-180)-MycHis, AIRE-(181–545)-MycHis, AIRE-(1-343)-MycHis, and AIRE-(344–545)-MycHis. The human *CACYBP* gene was also cloned in pcDNA3.1 with a Flag tag. The human *AIRE* gene without any tag was also cloned in pcDNA3.1 (RRID: Addgene_138209).

### Thymus samples, cell culture, and transfection

Thymus samples were obtained from children who underwent corrective heart surgery in the Department of Cardiac Surgery, Hospital Universitari Vall d’Hebron Samples were cut into small pieces of about 1 cm^3^ and frozen at −80 °C. Samples were obtained after informed consent from the participants’ families, and the experimental procedures were approved by the local ethical review boards of participating institution (ref PR AG-145/2011) in line with the principles of the Declaration of Helsinki.

Human Embryonic Kidney 293 (HEK-293; RRID: CVCL_0045) were used [32]. Cells were cultured in D-MEM (Gibco - ThermoFisher, Waltham, MA, USA) supplemented with 10% fetal bovine serum (FBS, Gibco) and cultured at 37 °C with 5% CO_2_. Cells were authenticated by HLA typing, and regular absence of mycoplasma contamination was performed.

For transfection, approximately 2 × 10^5^ cells were plated in each well of 6-well plates (Corning™) with 2 ml of DMEM 10% FBS. After 24 h at 37 °C and 5% CO_2_ and 30 min prior to transfection, cells were serum-deprived by replacing the medium with 2 ml of non-supplemented DMEM. Transfection was prepared by diluting 1 µg DNA in non-supplemented DMEM culture medium and, separately, the required volume of polyethyleneimine (PEI, PolysciencesTM) in non-supplemented DMEM, with a PEI: DNA ratio of 3:1. After brief shaking, the volume of PEI transfection solution was applied dropwise to the volume of the transfection solution with the DNA constructs. The transfection solutions were kept at room temperature, mixed periodically every 5 min for 30 min, and then applied dropwise to each well. Cells were incubated for 4 h at 37 °C, and then the culture medium was removed and replaced by fresh DMEM supplemented with 10% FBS. After 48 h, cells were recovered either mechanically or by trypsinization.

To generate stable transfectants, after 48 h of transfection, medium was replaced by DMEM 10% FBS and 1 mg/ml G418 and incubated for 2–3 weeks. The expression level of genes of interest was periodically checked by flow cytometry. In cases in which more than a single positive population was detected, limiting dilution was performed. The presence of a unique positive population was confirmed by flow cytometry.

### Flow cytometry

About 5 × 10^5^ cells were trypsinized, plated in 96-well plates, and fixed with PFA 3.7% for 20 min at room temperature (RT). Cells were rinsed twice with PBS and then permeabilized with staining buffer (SB: PBS, 0.5% Triton X-100, and 2% FBS) and incubated for 1 h with primary antibodies diluted 1/100 in SB. Cells were washed three times with SB and incubated with secondary antibody at a 1/200 dilution for 1 h. After three washing steps, cells were then resuspended in PBS, and data were collected with a FACSCalibur or a FACSCanto cytometer (BD FACSCalibur Flow Cytometry System (RRID: SCR_000401)) and analyzed by FlowJo_V10 software.

### Western blot

Western blot was performed as previously described [33]. Twenty µg of cell lysates were loaded in 12% SDS-PAGE gels, and proteins were subsequently transferred to polyvinylidene fluoride (PVDF) membranes. PVDF membranes were blocked for 1 h with 5% skimmed milk in 0.1% Tween-20 in PBS (T-PBS). Membranes were incubated for 1 h with the corresponding primary antibodies, washed three times with T-PBS, incubated for 1 h with peroxidase-labeled secondary antibodies, and washed 3 times with T-PBS. Vinculin (VNC) was selected to compare the abundance of different proteins in cell lysates, as it was usually used as a housekeeping protein for loading control. Detection was performed by luminescence using Clarity Western ECL Blotting Substrate (Bio-Rad).

### Immunofluorescence

A total of 2 × 10^5^ cells were seeded in 24-well plates and cultured for 24 h. The cells were washed three times with PBS and fixed in 3.7% formaldehyde for 20 min at room temperature (RT). After fixation, cells were washed and permeabilized with SB for 5 min. They were then incubated for 1 h with the primary antibody (1:200 in SB), followed by three washes with SB and a 1 h incubation with the secondary antibody (1:400 in SB). Finally, the cells were washed three times with SB, twice with PBS, and mounted using ProLong Gold Antifade Mountant with DAPI (Thermo Fisher Scientific). Images were acquired using an Olympus Fluoview 1000 confocal microscope.

### Co-immunoprecipitation assay

Transiently transfected cells were treated with 20 µM MG132 for 4 h, collected, and washed twice with cold PBS. Cells were fixed in 500 µl of 0.37% formaldehyde in PBS for 10 min at RT. The fixation was quenched by adding 500 µl of cold PBS containing 1.25 M glycine (Sigma-Aldrich™), followed by centrifugation at 6,000 × g for 10 min at 4 °C. Pellets were washed with 1 ml of cold PBS and resuspended in 200 µl of cold immunoprecipitation lysis buffer (IP lysis buffer: 50 mM Tris-HCl pH 7.5, 150 mM NaCl, 1% NP40, 0.1% SDS, 1 mM EDTA, supplemented with cOmplete™ protease inhibitor cocktail (Roche)). After a 1-h incubation on ice, samples were sonicated (three 10-sec cycles at 60 Hz). Simultaneously, anti-c-Myc (Pierce) or anti-Flag (Sigma-Aldrich Cat #M8823, RRID: AB_2637089) magnetic beads were equilibrated and kept on ice. Lysates were centrifuged at 13,000 x g for 10 min at 4 °C, and the supernatants were collected. A 20-µl aliquot of each sample was reserved as the total lysate (input), mixed with 20 µl of RBS, and stored at −20 °C. The remaining volume was incubated with the magnetic beads for 4 h at 4 °C under rotation. The beads were then washed once with 300 µl of IP lysis buffer and three times with 300 µl of cold IP wash buffer (IPW buffer: 50 mM Tris-HCl pH 7.5, 150 mM NaCl, 0.05% NP40, 1 mM EDTA, with protease inhibitors) using a magnetic separator. Finally, the beads were resuspended in 40 µl of RBS, boiled for 15 min, and analyzed by Western blot.

For ex vivo experiments, thymic tissues were mechanically disrupted using a Potter-Elvehjem homogenizer in ice-cold isotonic lysis buffer (20 mM Tris-HCl pH 8.0, 150 mM NaCl, with protease inhibitors). The homogenate was sonicated at 25 Hz for 10 min (1 s on / 5 s off) on ice and centrifuged at 13,000 × g for 10 min at 4 °C. Protein concentration in the supernatant was determined using a BCA kit (Thermo Fisher). While 10 µg of total protein was reserved as input, approximately 1 mg was used for the co-immunoprecipitation assay. Lysates were pre-cleared with 25 µL of protein G-Sepharose for 1 h under rotation, centrifuged at 6,000 x g for 2 min at 4 °C, and transferred to new tubes. Samples were incubated with 10 µg of primary antibodies for 2 h under rotation, followed by an overnight (O/N) incubation with 25 µL of protein G-Sepharose. The beads were washed five times with 300 µL of ice-cold isotonic lysis buffer (6,000 x g, 2 min, 4 °C). Finally, the protein complexes were resuspended in 40 µL of RBS, boiled for 5 min, and analyzed by Western blot.

### Modeling of SIAH1 interactions with CACYBP and AIRE motifs

To model the complexes between SIAH1 (residues 125–282) and peptides from SIP (residues 58–70) or AIRE (residues 117–129 and 414–426, modeled as independent complexes), AlphaFold v2.2.4 [34] was employed. The software was installed and executed within a Docker container, with the source code and required databases (full versions) downloaded on October 18, 2022, from the official repository (https://github.com/deepmind/alphafold). AlphaFold was run using the “multimer” model with default parameters; multiple sequence alignments were constructed using all templates available at the time of download. For each complex, 24 models were generated, and the one with the highest ranking score was selected for further analysis.

### Apoptosis assays

HEK-293 cells (RRID: CVCL_0045) or AIRE-transfected variants were seeded at a density of 1 × 10^5^ cells per well. After 48 h of incubation at 37 °C, the cells were treated with either DMSO (as a control for basal apoptosis) or 50 µM etoposide (Sigma-Aldrich™) to induce apoptosis, followed by an additional 24 h incubation. Apoptosis was assessed using an Annexin V-Alexa Fluor 488 and Propidium Iodide (PI) Detection Kit (Thermo Fisher Scientific™) according to the manufacturer’s instructions. Samples were subsequently analyzed by flow cytometry.

### Ubiquitination assays

Stable clones expressing AIRE-Myc/His, AIRE-(1-343)-Myc/His, or AIRE-(181–545)-Myc/His were transiently transfected with Flag-SIAH1 and Ub-Flag constructs and treated with 20 µM MG132 (Sigma-Aldrich) for 4 h at 37 °C. Cells were then harvested and lysed in lysis buffer (LB: 50 mM Tris-HCl pH 7.5, 150 mM NaCl, 0.5% NP40, 1% Triton X-100, supplemented with cOmplete™ protease inhibitor cocktail) for 1 h at 4 °C. Lysates were homogenized by passing the samples several times through a 30-gauge needle. AIRE-Myc/His proteins were immunoprecipitated using anti-Myc magnetic beads (Thermo Fisher Scientific Cat# 88843, RRID: AB_2861398) as described above. Proteins were resolved by 12% SDS-PAGE and transferred onto PVDF membranes. Ubiquitinated proteins were detected using a rabbit polyclonal anti-ubiquitin antibody (Sigma-Aldrich Cat# U5379, RRID: AB_477667).

### AIRE degradation assays

Approximately 2 × 10^5^ HEK293 cells (RRID: CVCL_0045) or stable AIRE-expressing cells were seeded in 6-well plates (Corning™) in 2 ml of DMEM supplemented with 10% FBS. After 24 h at 37 °C, cells were transiently transfected as described above with AIRE-Myc/His, AIRE-V3E-Myc/His, AIRE-P4E-Myc/His, Flag-SIAH1, or Flag-∆SIAH1 (a catalytic domain-deficient mutant). Cells were harvested by scraping, washed twice with ice-cold PBS, and lysed in lysis buffer (LB) for 1 h on ice. Lysates were homogenized by passing them several times through a 30-gauge needle. Supernatants were then collected by centrifugation at 13,000 x g for 10 min at 4 °C. Protein concentrations were determined, and samples were analyzed by SDS-PAGE and Western blot. Densitometric analysis was performed using Fiji/ImageJ software (RRID: SCR_003070).

### Statistical analysis

Statistical analysis was performed using GraphPad Prism 8 software. The Mann-Whitney *U* test (*p* < 0.05, *p < 0.01, **p < 0.001, ***p < 0.0001) was used to compare the corrected total cell fluorescence (CTCF) between control and AIRE-transfected cells. The Wilcoxon signed-rank test (*p* < 0.05, *p < 0.01, **p < 0.001, ***p < 0.0001) was employed to compare densitometric values of protein levels between the control and transfected groups.

## Results

### CACYBP/SIP levels are upregulated in AIRE-expressing cells

**Fig. 1:**
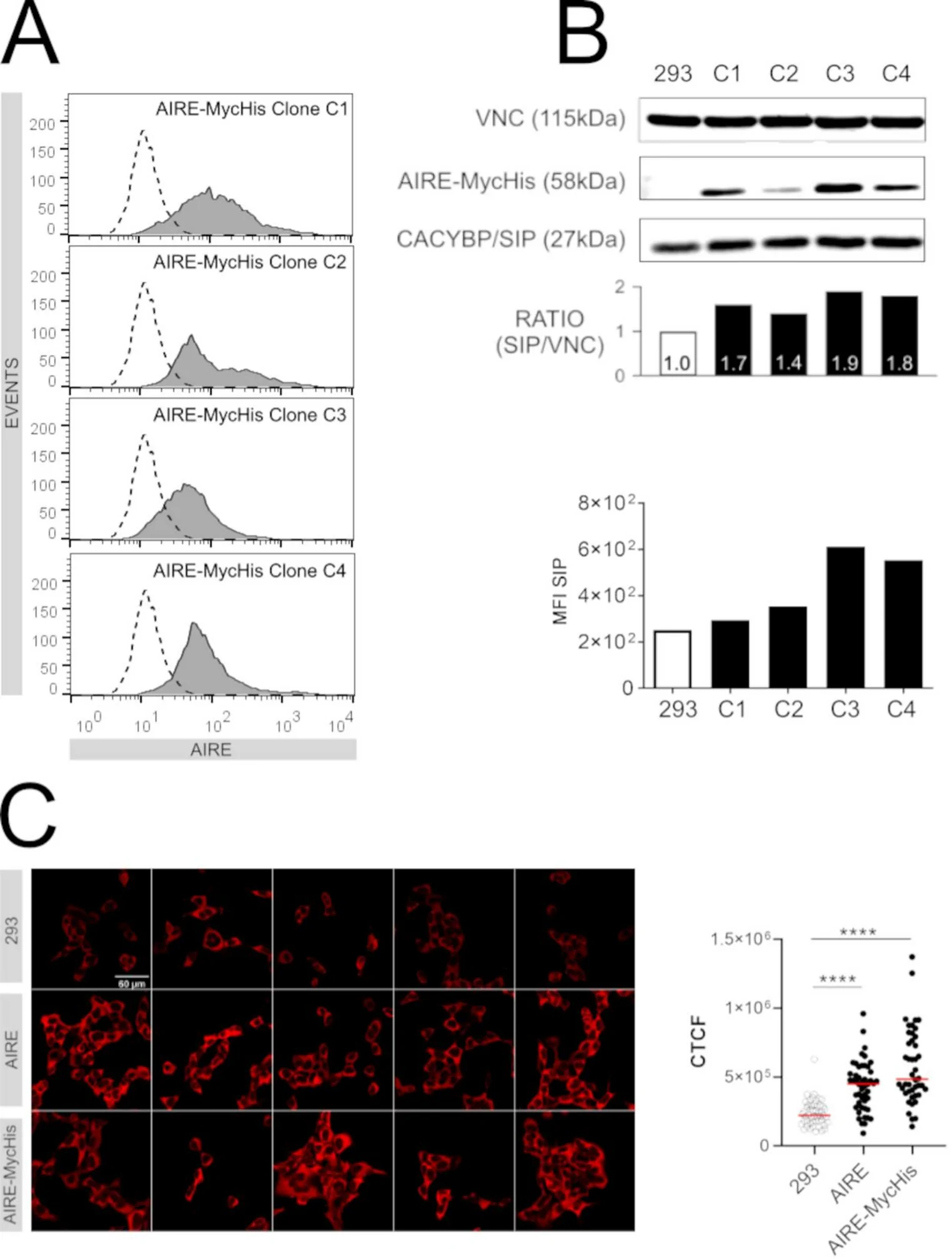
CACYBP/SIP levels are upregulated in AIRE-expressing cells. **A** Flow cytometry analysis of HEK-293 clones stably transfected with AIRE-MycHis. Protein expression was detected using an anti-Myc antibody. Dashed line: parental HEK-293; filled histogram: AIRE-transfected clones. **B** Analysis of SIP protein expression in AIRE⁺ clones. Upper panel: Representative Western blot of SIP levels. The bar graph represents the SIP/vinculin ratio determined by densitometric analysis, normalized to parental HEK-293 cells. Lower panel: Mean fluorescence intensity (MFI) quantified by flow cytometry of SIP expression in HEK-293 cells and AIRE-expressing clones (C1–C4, as described in panel A). In the figure a representantive experiment is shown, but 3 different experiments were made, and SIP was increased in all clones. **C** Confocal immunofluorescence microscopy of SIP expression in parental HEK-293, HEK-293-AIRE (untagged), and HEK-293-AIRE-MycHis. Cells were fixed and stained with an anti-SIP antibody (red). Micrographs were acquired at 60× magnification. Right panel: Quantification of Corrected Total Cell Fluorescence (CTCF) from 50 randomly selected cells per group. CTCF was calculated as: Integrated Density – (Area of selected cell × Mean fluorescence of background readings). Statistical significance was determined using the Mann-Whitney U test (*P < 0.05, **P < 0.01, ***P < 0.001, ****P < 0.0001)

We previously characterized the proteomic profiles of HT93 epithelial cells (thyroid origin) and AIRE-transfected HT93 cells, demonstrating that AIRE^+^ cells exhibit increased apoptosis. Additionally, higher levels of SIP were detected in AIRE^+^ cells compared to control groups. To verify that this effect is mediated by AIRE expression and is not cell-line specific, we generated a stable HEK-293 transfectant overexpressing AIRE with a C-terminal Myc/His tag (AIRE-MycHis). Four AIRE^+^ clones were isolated via limiting dilution (Fig. 1A). Increased SIP protein expression was confirmed across all four clones using Western blot, flow cytometry (Fig. 1B), and immunofluorescence (IF, Fig. 1C). To exclude potential tag-induced artifacts, a tag-free AIRE transfectant was also generated. IF analysis revealed SIP elevation levels comparable to the AIRE-MycHis clones (Fig. 1C). These findings, consistent with our prior observations in HT93 cells, demonstrate that AIRE expression increases SIP protein abundance.

To determine whether AIRE modulates SIP through transcriptional induction, total RNA from HEK-293 and AIRE⁺ clones was purified and reverse-transcribed into cDNA. PCR analysis of CACYBP mRNA transcripts revealed higher transcript abundance in all AIRE^+^ clones (Figure S1).

Furthermore, we examined the reciprocal effect of SIP on AIRE expression. Human *CACYBP* (encoding SIP) was cloned with a Flag tag into pcDNA3.1 (SIP-Flag) and transiently transfected into AIRE-expressing HEK-293 clones. Western blot analysis showed an increase in AIRE levels in three SIP-Flag transfectants compared to non-transfected controls (Figure S2). This mutual upregulation of intracellular SIP and AIRE levels suggests a bidirectional regulatory mechanism between these two proteins.

### AIRE and SIP do not interact in transfected HEK-293 cells

**Fig. 2:**
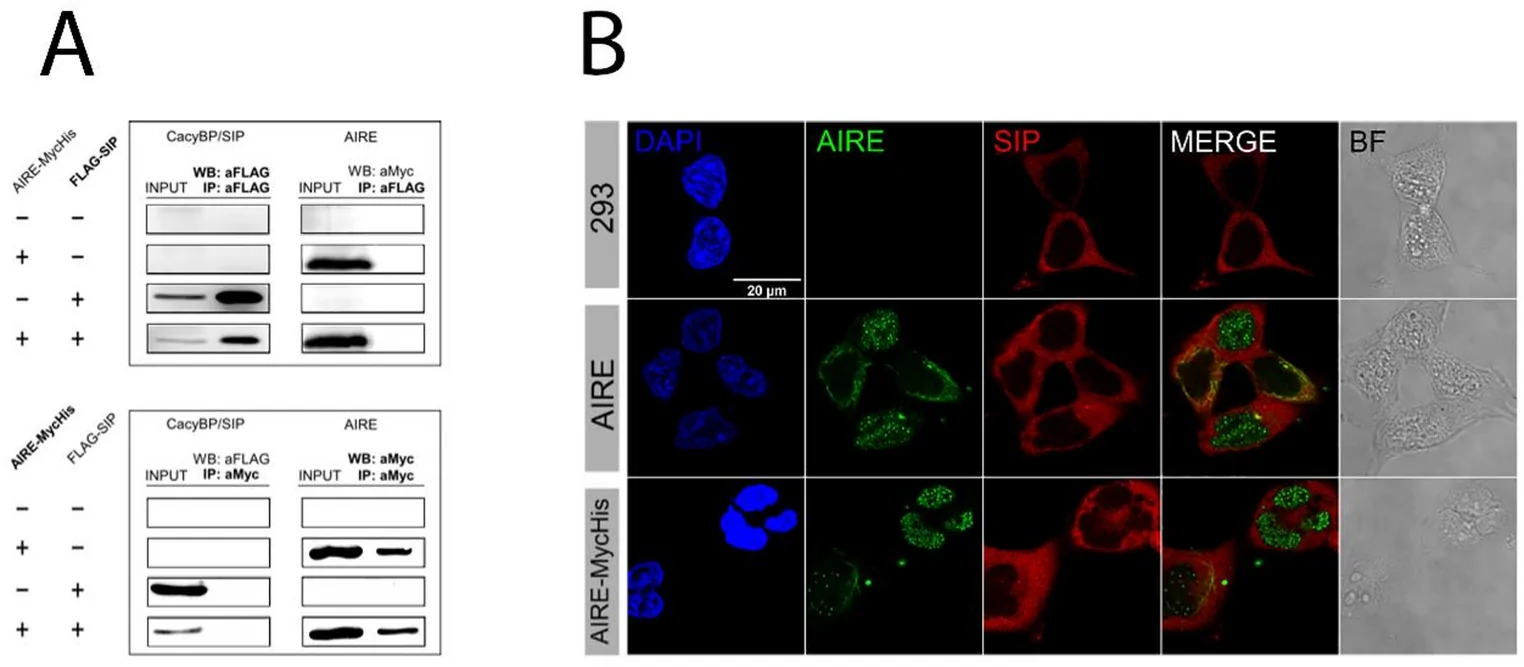
AIRE does not physically associate with SIP in transfected HEK-293 cells. **A** Co-immunoprecipitation (Co-IP) assay of HEK-293 cells transiently co-transfected with AIRE-MycHis and SIP-Flag. Lysates were subjected to immunoprecipitation (IP) using anti-Flag or anti-Myc antibodies, followed by Western blot (WB) analysis for the indicated tags. INPUT: Total cell lysate (1/100 of the starting material). No reciprocal interaction was detected between AIRE and SIP. **B** Confocal immunofluorescence microscopy of HEK-293 cells stably expressing either untagged AIRE or AIRE-MycHis. Fixed cells were stained with anti-AIRE (green) and anti-SIP (red) antibodies; DAPI (blue) was used for nuclear counterstaining. Merged images (green and red channels) and brightfield (BF) micrographs are shown. No intracellular co-localization between AIRE and SIP was observed. Images were acquired at 60× magnification with a 4× digital zoom

To investigate a potential direct interaction between AIRE and SIP in vivo, co-immunoprecipitation (Co-IP) assays were performed. HEK-293 cells were co-transfected with AIRE-MycHis and SIP-Flag constructs and subsequently treated with the proteasome inhibitor MG-132 for 4 h. Following incubation, AIRE was immunoprecipitated using an anti-Myc monoclonal antibody (mAb), and the presence of both proteins was assessed via immunoblotting with specific anti-Myc and anti-Flag antibodies. Reciprocal Co-IP experiments were also conducted by immunoprecipitating SIP with an anti-Flag mAb, followed by Western blot analysis for Flag and Myc. As shown in Fig. 2A, no physical interaction was detected between AIRE and SIP under these conditions. Furthermore, confocal microscopy was employed to evaluate intracellular localization in HEK-293 cells stably expressing AIRE; however, no significant co-localization was observed (Fig. 2B).

### AIRE interacts with SIAH1 and SIAH2 in transfected HEK-293 cells and in the thymus

**Fig. 3:**
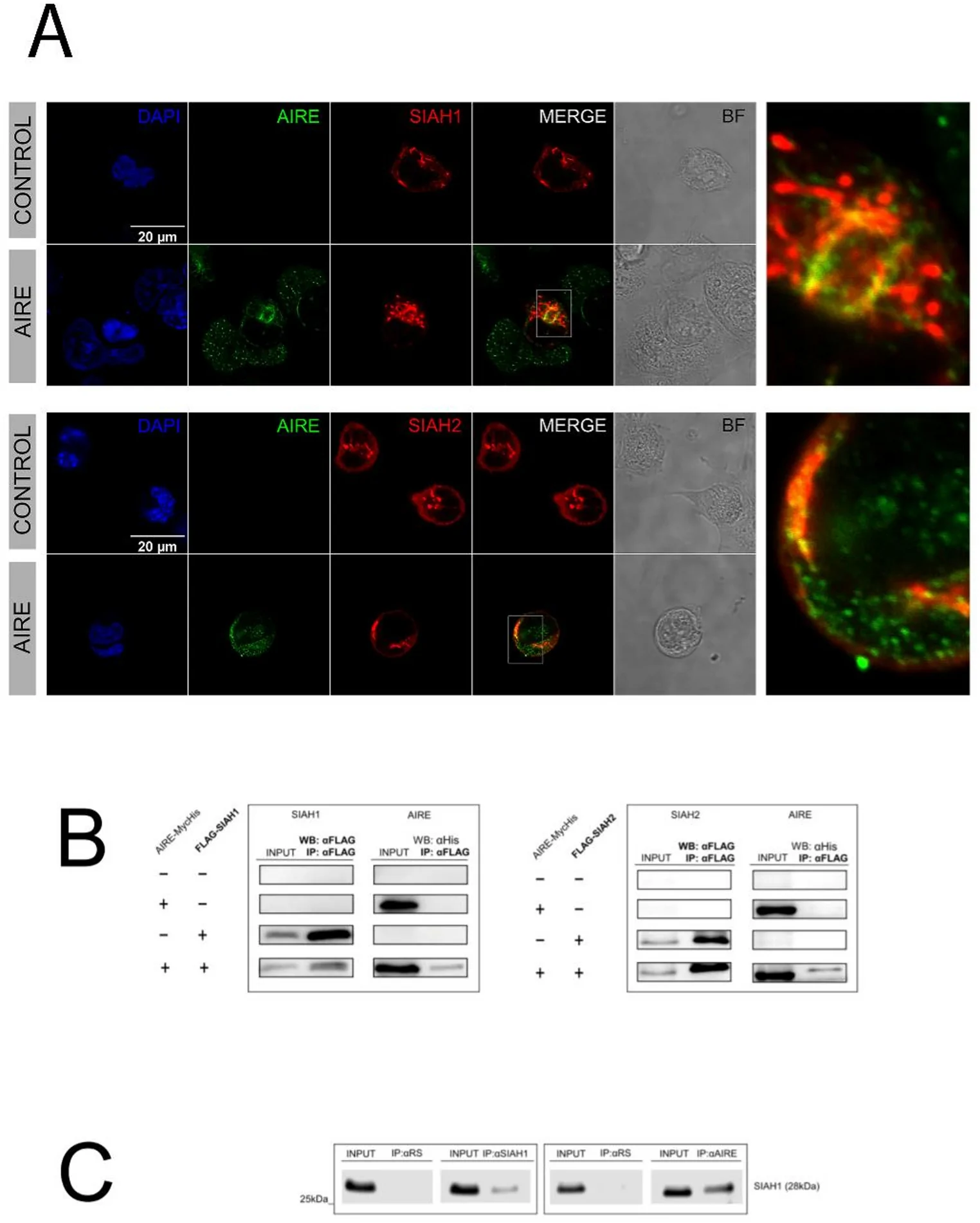
AIRE co-localizes and interacts with SIAH1 and SIAH2. **A** Confocal immunofluorescence analysis of parental HEK-293 cells or HEK-293 cells stably expressing AIRE, transiently transfected with Flag-SIAH1 or Flag-SIAH2. Fixed cells were stained with anti-AIRE (green) and anti-Flag (red) antibodies; DAPI (blue) was used for nuclear counterstaining. Merged images (green and red channels) and brightfield (BF) micrographs are shown. Intracellular co-localization between AIRE and SIAH1/2 was observed in the cytoplasm. Images were acquired at 60× magnification with a 4× digital zoom. **B** Co-immunoprecipitation (Co-IP) assay of HEK-293 cells transiently co-transfected with AIRE-MycHis and Flag-SIAH1 or Flag-SIAH2. SIAH proteins were immunoprecipitated using an anti-Flag antibody, followed by Western blot (WB) analysis for Flag or His tags. INPUT: Total cell lysate (1/100). **C** Endogenous interaction in human thymus. Thymus tissue samples were homogenized (potterized), and protein extracts were subjected to IP using normal rabbit serum (αRS) as a negative control, a rabbit anti-SIAH1 polyclonal antibody αSIAH1), or a rabbit anti-AIRE polyclonal antibody αAIRE). Following immunoprecipitation, SIAH1 was detected by WB. INPUT: Total thymus lysate (1/100)

Since SIP serves as an adaptor protein for the E3 ubiquitin ligases SIAH1 and SIAH2, we employed confocal microscopy to investigate the potential co-localization of AIRE with these ligases. HEK-293 cells stably expressing AIRE were transiently transfected with SIAH1 or SIAH2 constructs. As shown in Fig. 3A, AIRE co-localized with both SIAH1 and SIAH2 within the cytoplasm. The expression of AIRE outside the nucleus was in fibrillar structures, what has been previously reported in our laboratory [21].

SIAH proteins recognize a consensus binding motif, PxAxVxP (where x represents any amino acid). In silico analysis of the AIRE primary sequence identified two putative SIAH-interacting motifs (SIMs): a canonical PKALVPP sequence (residues 119–125, SIM1) and a non-canonical PLLCVGP sequence (residues 416–422, SIM2), the latter featuring a leucine substitution for alanine at the second position (Figure S3).

To evaluate the physical association between AIRE and SIAH1, Co-IP assays were performed using AIRE-MycHis and Flag-SIAH1 constructs. Following immunoprecipitation of AIRE with an anti-Myc mAb, Flag-SIAH1 was detected via Western blot (Fig. 3B), confirming an interaction between AIRE and SIAH1 in transfected HEK-293 cells. Analogous experiments using Flag-SIAH2 yielded comparable results (Fig. 3B), indicating that AIRE also associates with SIAH2.

Finally, to validate this interaction in a physiological context, Co-IP was performed using human thymus tissue lysates. Following AIRE immunoprecipitation, SIAH1 was recovered and detected by Western blot (Fig. 3C), demonstrating that the AIRE-SIAH1 interaction occurs endogenously within the human thymus.

### AIRE interacts with SIAH proteins via its N-terminal SIAH-binding motif

**Fig. 4:**
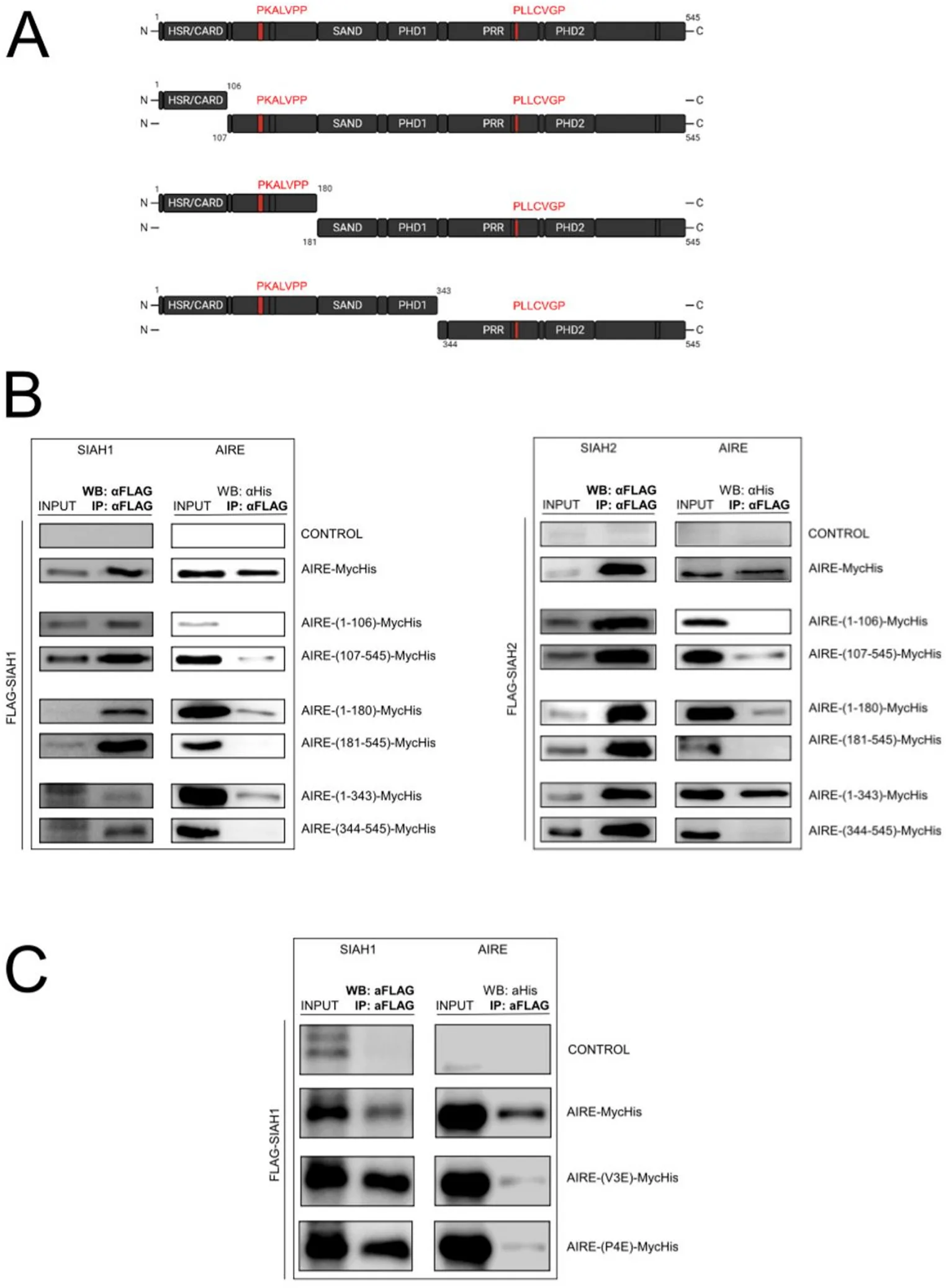
AIRE interacts with SIAH1 and SIAH2 via the first SIAH-interacting motif (SIM1). **A** Schematic representation of full-length AIRE and various truncation constructs. The two putative SIAH-interacting motifs (SIM1 and SIM2) are highlighted in red. **B** Co-immunoprecipitation (Co-IP) assay in HEK-293 cells transiently co-transfected with Flag-SIAH1 or Flag-SIAH2 and either full-length or truncated AIRE-MycHis constructs. SIAH proteins were immunoprecipitated using an anti-Flag antibody, followed by Western blot (WB) analysis for Flag and His tags. INPUT: Total cell lysate (1/100). **C** Site-directed mutagenesis analysis. HEK-293 cells were co-transfected with Flag-SIAH1 and either wild-type AIRE-MycHis or the point mutants V123E (AIRE-V3E) and P125E (AIRE-P5E) within the first motif. Following anti-Flag immunoprecipitation, AIRE recovery was assessed by anti-His WB. INPUT: Total cell lysate (1/100)

To map the specific AIRE regions mediating the interaction with SIAH proteins, several C-terminally Myc/His-tagged AIRE truncation mutants were cloned into the pcDNA3.1 expression vector. These constructs were designed to include or exclude the two putative SIAH-interacting motifs: (1) Fragment 1–106 (CARD domain, lacking both SIMs); (2) Fragment 107–545 (containing both SIMs and the SAND, PHD1, PRR, and PHD2 domains); (3) Fragment 1–180 (CARD and SIM1); (4) Fragment 181–545 (SAND, PHD1, PRR, PHD2, and SIM2); (5) Fragment 1–343 (CARD, SAND, PHD1, and SIM1); and (6) Fragment 344–545 (PRR, PHD2, and SIM2) (Fig. 4A).

Co-transfection assays with Flag-SIAH1 revealed that only the full-length AIRE and constructs containing SIM1 (107–545, 1–180, and 1–343) were recovered after anti-Flag immunoprecipitation. In contrast, constructs lacking this motif (1–106, 181–545, and 344–545) showed no interaction with SIAH1 (Fig. 4B). Equivalent results were obtained in experiments with SIAH2 (Fig. 4B).

To further validate the requirement of SIM1, site-directed mutagenesis was performed to replace Val123 and Pro125 with Glutamic acid (V123E and P125E). Co-IP analysis showed a marked reduction in AIRE recovery following SIAH1 immunoprecipitation for both mutants (Fig. 4C), confirming that the motif spanning residues 119–125 is the primary SIAH-binding site.

Finally, we performed a competitive binding assay to determine if AIRE and SIP compete for SIAH1 association. Recombinant StII-∆SIAH1 (a catalytically inactive protein, lacking N-terminal residues 1–89) was incubated with HEK-293-AIRE cell lysates in the presence or absence of purified SIP-His protein. The addition of SIP significantly reduced the amount of AIRE recovered, suggesting that AIRE and SIP compete directly for the same binding site on SIAH1 (Figure S4).

### Structural modeling predicts that SIAH1 selectively interacts with the AIRE_119-125_ motif

**Fig. 5:**
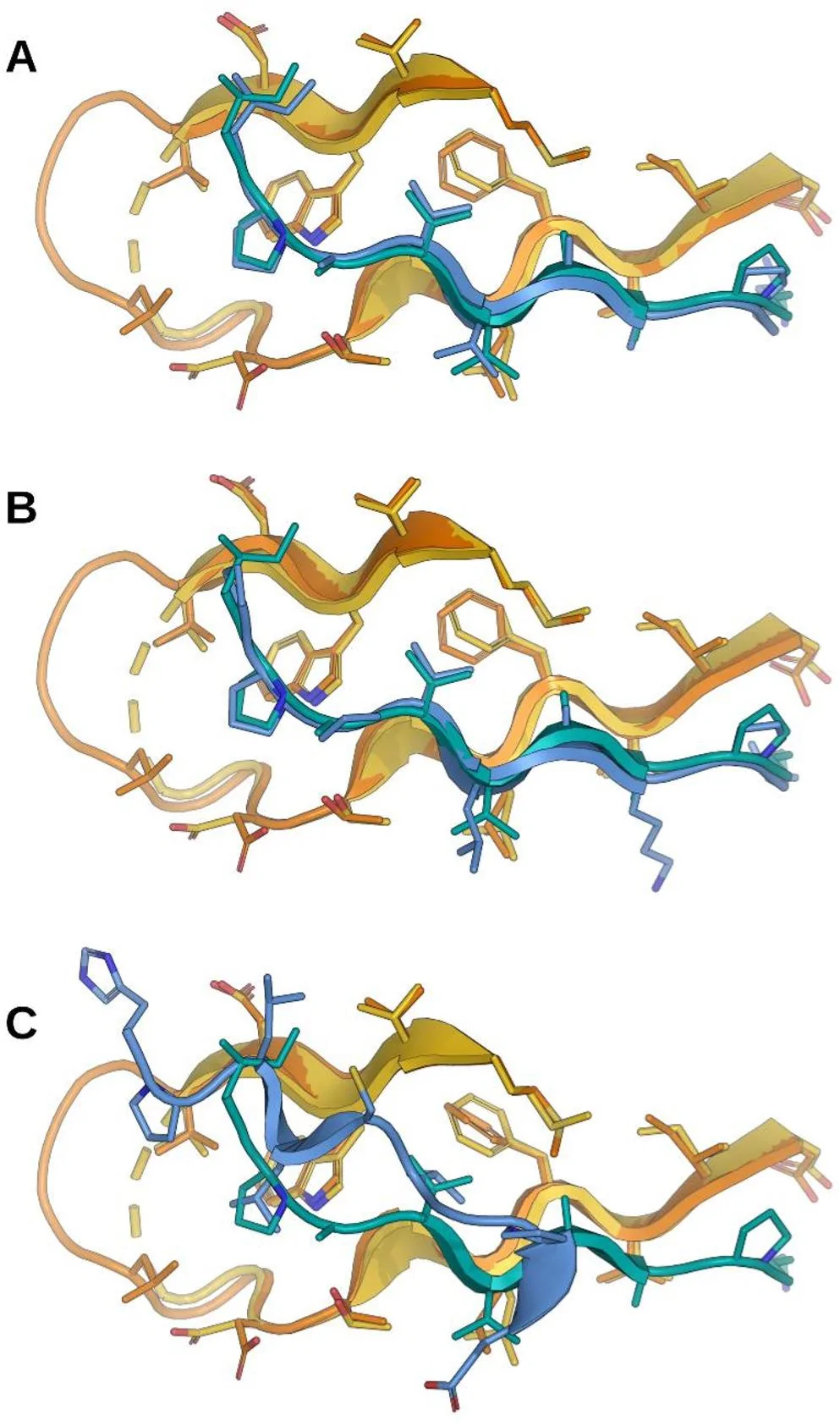
Structural characterization of SIAH1-peptide complexes at the binding interface. The panels illustrate the structural superposition of the crystallographic SIAH1–SIP_59-67_ complex (PDB 2A25) with the complexes modeled by AlphaFold: **A** SIAH1–SIP_58-70_, **B** SIAH1–AIRE_117-129_, and **C** SIAH1–AIRE_414-426_. Color code: Gold: crystallographic SIAH1 structure (dashed lines indicate unresolved loops); Green: crystallographic SIP_59-67_ peptide; Orange: AlphaFold-predicted SIAH1 structure; Blue: AlphaFold-predicted peptide structure. Structural representation: Both ribbon diagrams (highlighting β-strand secondary structures) and stick representations (side chains) are shown. The models display SIAH1 residues 162–180, which constitute the primary binding surface, and nine residues from each peptide corresponding to the crystallographically resolved region. Sequences are as follows (core binding residues in bold): KPAAVVAPI (SIP_59-67_), VPKALVPPP (AIRE_118-126_), and HPLLCVGPE (AIRE_415-423_). Note that the AIRE_415-423_ sequence adopts an inverted orientation within the binding pocket relative to the canonical SIP_59-67_ and AIRE_118-126_ binding modes

To assess the structural basis of the AIRE-SIAH1 interaction, we performed in silico modeling using AlphaFold. We first validated the model by superimposing the predicted SIAH1–SIP peptide complex with the existing crystallographic structure (PDB 2A25). Subsequent modeling was conducted for the two putative SIMs of AIRE. As shown in Fig. 5A, the modeled SIAH1–SIP_58-70_ complex aligns precisely with the experimental crystal structure. Similarly, the SIAH1–AIRE_117-129_ complex exhibited an identical binding mode, overlapping perfectly with the SIAH1–SIP_58-70_ interface (Fig. 5B). In contrast, the SIAH1–AIRE_414-426_ peptide appeared significantly less structured and adopted an inverted orientation (Fig. 5C), suggesting a lack of stable interaction with SIAH1.

Model reliability was further evaluated using AlphaFold confidence metrics (Figure S5A (SIAH1-SIP_58-70_), S5B (SIAH1-AIRE_117-125_) and S5C (SIAH1-AIRE_414-426_)). The pLDDT (per-residue confidence score) for AIRE_414-426_ was below 50, a threshold indicative of disordered regions [35]. Conversely, pLDDT values exceeded 90 for the core residues of both SIP_58-70_ and AIRE_117-129_, predicting high structural accuracy. Furthermore, Predicted Aligned Error (PAE) matrices confirmed high confidence for the interchain interactions in the SIP and AIRE complexes, while confidence for the AIRE_414-426_ interaction remained low.

Both SIP_58-70_ and AIRE_117-129_ formed a parallel -sheet with SIAH1 residues Val164–Thr168, mediated by residues Pro60–Val64 in SIP, seen in the crystal (Figure S6A) and with AlphaFold (Figure S6B) and Pro119–Val123 in AIRE (Figure S6C). Although AIRE_414-426_ contains a potential SIM (PxxxVxP), we hypothesized that the substitution of the canonical Ala62 (found in SIP) with a bulky Leu418 in AIRE results in steric hindrance with SIAH1 residues Thr156 and Met180. To test this, we re-evaluated the AIRE_414-426_ model after introducing a L418A mutation. The AlphaFold prediction confirmed that this single substitution restores the canonical binding pose observed in SIP_58-70_ and AIRE_117-129_ (Figure S7A), with a significant improvement in confidence scores (Figure S7B). These results demonstrate that an alanine at the second position of the motif is a critical determinant for SIAH1 binding.

### The CARD domain and the SIAH1-interacting motif are essential for AIRE-mediated apoptosis

Previous studies by our group and others have established that AIRE expression induces apoptosis. To determine whether the SIAH1-interacting motif (degron) is required for this proapoptotic activity, we analyzed stable transfectants expressing full-length AIRE (with or without a Myc/His tag) and four AIRE truncation mutants: AIRE_1-180_ (containing the CARD domain and the degron), AIRE_1-106_ (CARD domain only), AIRE_107-545_ (degron sequence without CARD), and AIRE_181-545_ (lacking both domains).

**Fig. 6:**
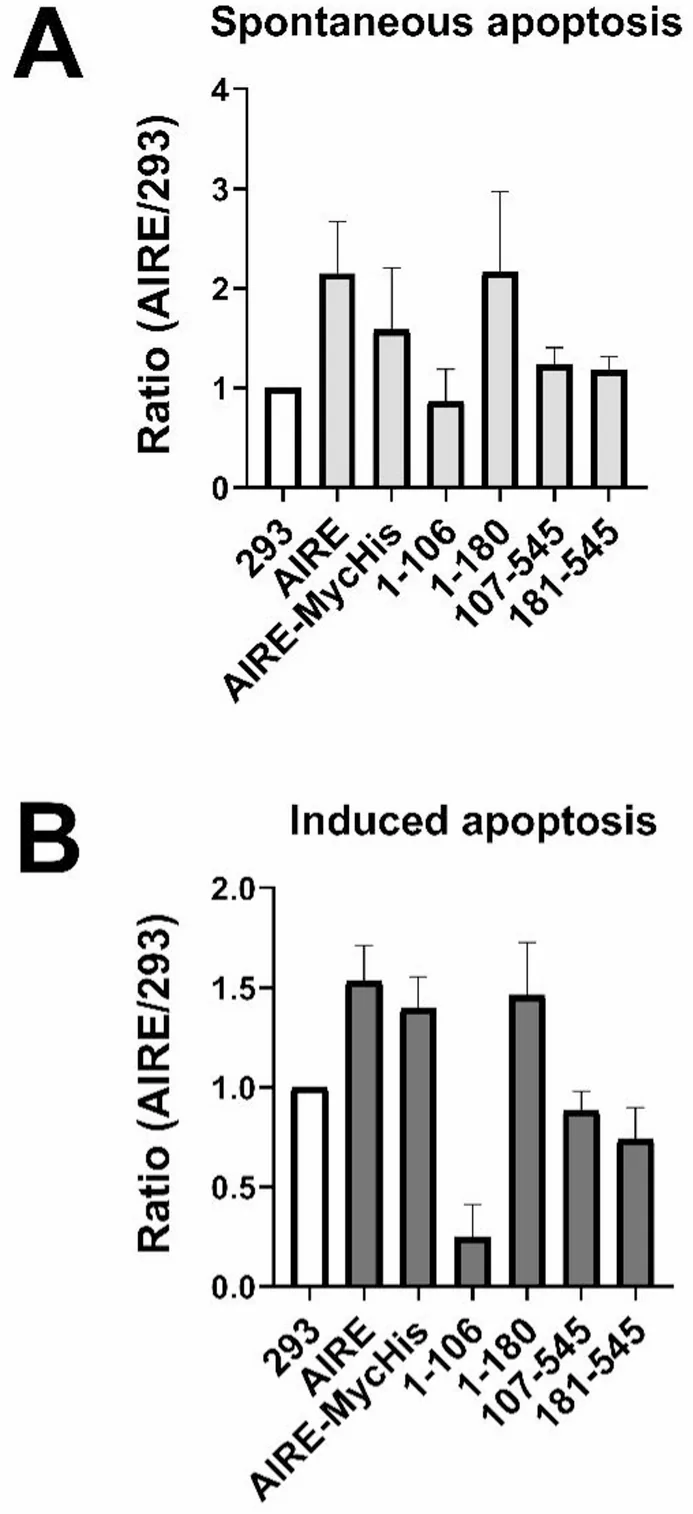
The CARD domain and the SIAH1-interacting motif (SIM1) of AIRE are essential for apoptosis induction in HEK-293 cells. Parental HEK-293 cells and various stable AIRE transfectants were treated with DMSO (vehicle control) to measure spontaneous apoptosis or with etoposide (50 µM) to evaluate chemically induced apoptosis. Apoptotic levels were assessed by flow cytometry using Annexin V-FITC and Propidium Iodide (PI) staining. Upper panel: Fold increase in spontaneous apoptosis of different stable AIRE transfectants relative to parental HEK-293 cells after 24 h. Lower panel: Fold increase in etoposide-induced apoptosis across the indicated cell lines after 24 h. Data represent the mean of *n* = 4 independent experiments (measures range, from 6 to 20). Statistical significance was determined using the Kruskal-Wallis test followed by post-hoc comparisons (*P < 0.05, **P < 0.01, ***P < 0.001, ****P < 0.0001). Values in (A) and (B) represent total apoptosis, calculated as the sum of early apoptotic (Annexin V^+^/PI^−^) and late apoptotic (Annexin V^+^/PI^+^) populations, normalized to parental HEK-293 controls

Cells were treated with DMSO to assess spontaneous apoptosis or with etoposide to evaluate sensitized apoptotic responses. Apoptosis levels were quantified by Annexin V staining and flow cytometry (Figure S8 shows a representative workflow of the flow cytometry analysis). A significant increase in spontaneous apoptosis (Fig. 6A) or etoposide-induced apoptosis (Fig. 6B) was observed only in cells expressing full-length AIRE or the AIRE fragment, which retains both the CARD domain and the SIM1. In contrast, constructs lacking either the CARD domain or the degron sequence failed to promote apoptosis, indicating that both regions are strictly required for AIRE’s proapoptotic function.

### SIAH1-mediated ubiquitination and proteasomal degradation of AIRE

**Fig. 7:**
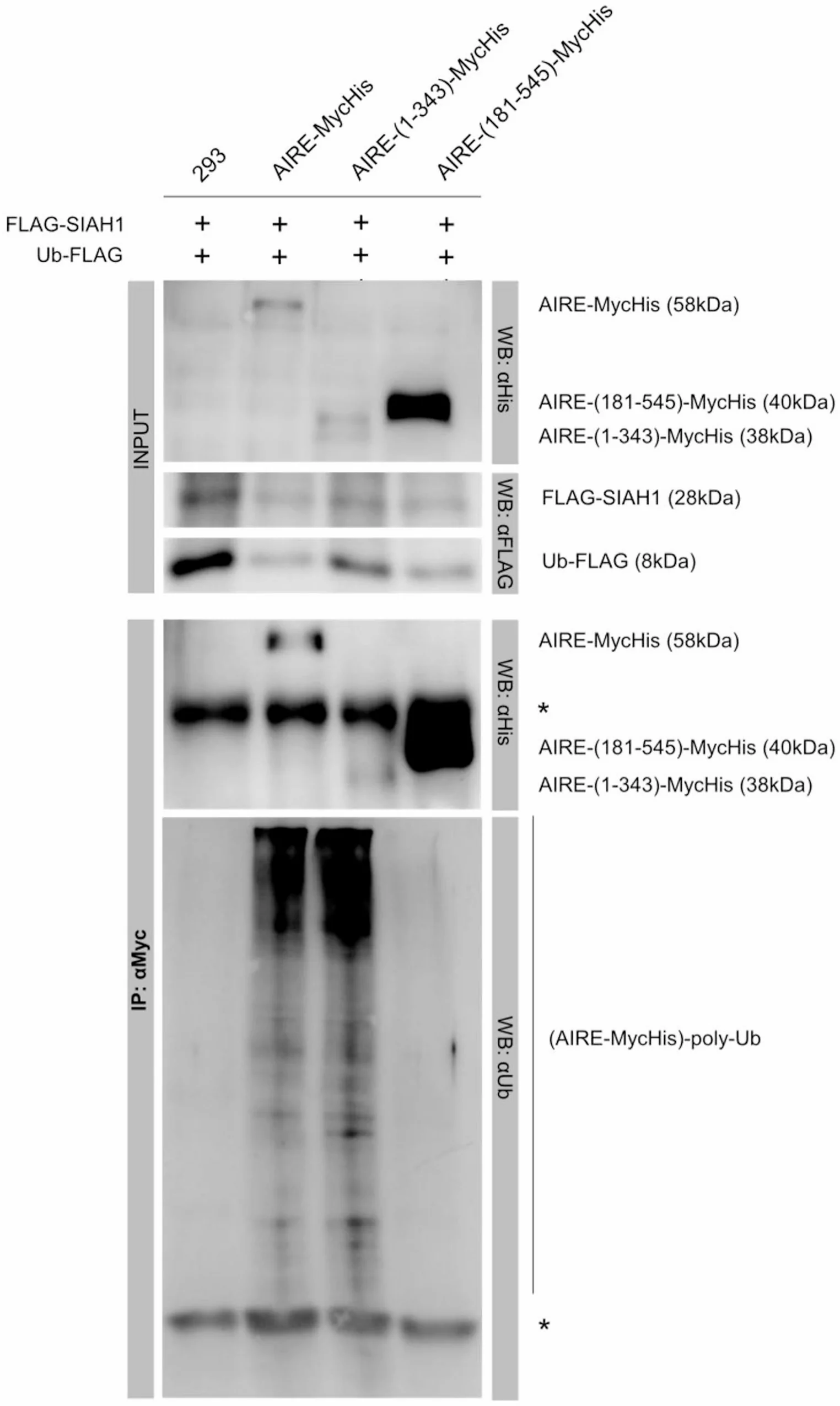
SIAH1 mediates the polyubiquitination of AIRE. Parental HEK-293 cells and stable transfectants expressing AIRE-MycHis, AIRE_1-343_-MycHis (containing the SIAH-interacting motif), or AIRE_181-545_-MycHis (lacking the motif) were transiently co-transfected with Flag-SIAH1/2 and Flag-Ubiquitin (Flag-Ub). After 48 h, proteasomal degradation was inhibited with MG-132 for 4 h. AIRE was immunoprecipitated (IP) using an anti-Myc antibody. AIRE proteins were detected by Western blot (WB) using an anti-His antibody. SIAH proteins and Flag-Ub were detected with an anti-Flag antibody, and polyubiquitinated AIRE species were visualized using an anti-Ub antibody. (*) Denotes the IgG heavy chain from the immunoprecipitating antibodies. VNC: Vinculin (loading control); β-CAT: β-catenin; β-ACT: β-actin

To determine if the physical association between AIRE and SIAH1 leads to post-translational modification, we performed ubiquitination assays in HEK-293 cells. AIRE-MycHis was co-transfected with Flag-SIAH1 and Flag-Ubiquitin (Flag-Ub). Additionally, cells stably expressing AIRE_1-343_ (containing SIM1) or AIRE_181-545_ (lacking the motif) were analyzed. Following immunoprecipitation of AIRE with an anti-Myc mAb, polyubiquitination was detected using a specific anti-Ub antibody. As shown in Fig. 7, robust polyubiquitination was observed for both full-length AIRE and the AIRE_1-343_ fragment, whereas no ubiquitination was detected in control HEK-293 cells or the AIRE_181-545_, confirming that the SIAH1-interacting motif is required for AIRE ubiquitination.

**Fig. 8:**
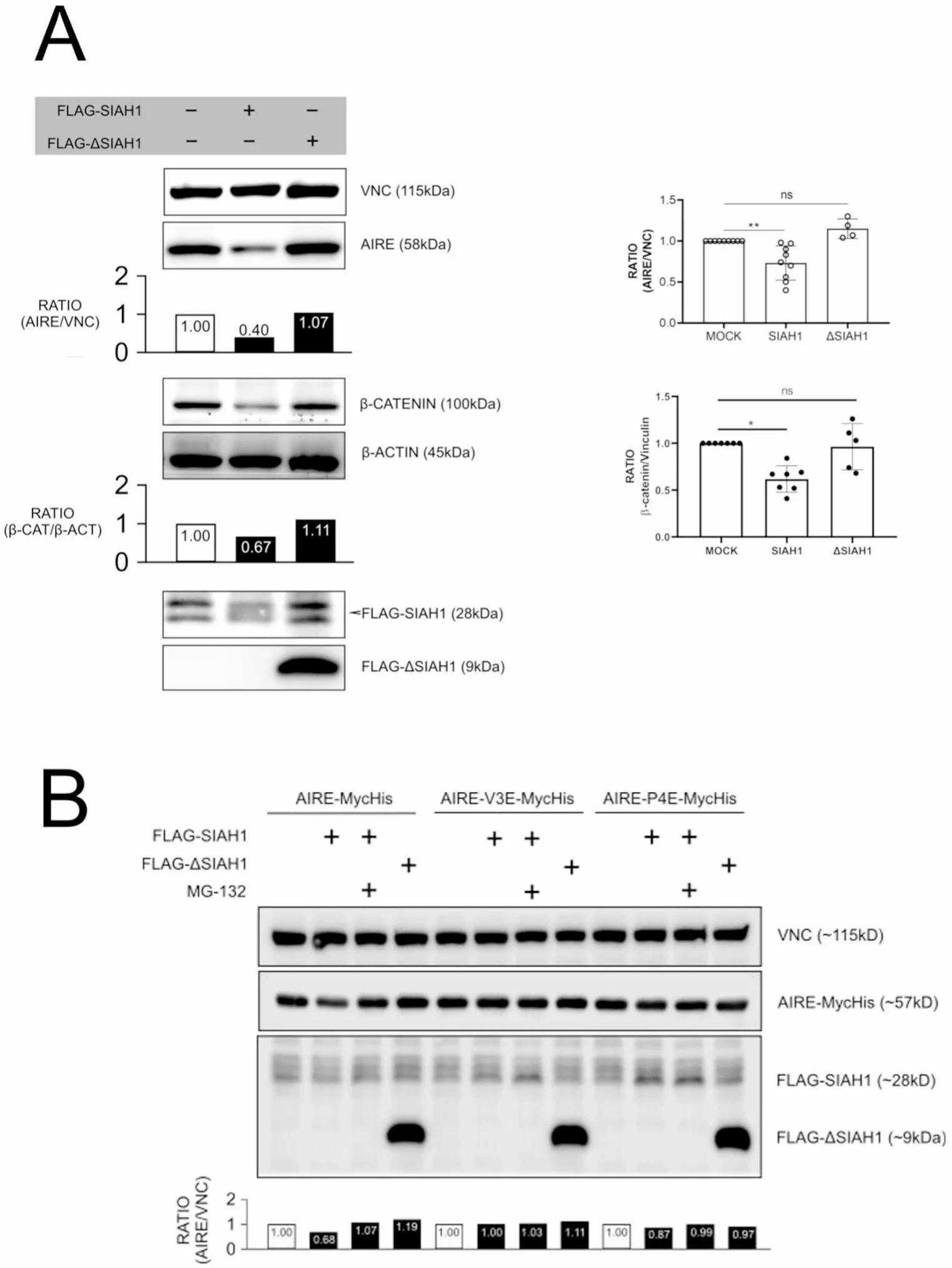
SIAH1 mediates the proteasomal degradation of AIRE. **A** HEK-293 cells stably expressing AIRE were transiently transfected with SIAH1 or a catalytically inactive protein, ΔSIAH1 (lacking residues 1–89). Protein levels were assessed by Western blot (WB). Bar graphs represent the AIRE/Vinculin or β-catenin/Vinculin densitometric ratios. Right panel: Normalized values for AIRE/Vinculin ratios in non-transfected (*n* = 9), SIAH1-transfected (*n* = 9), and ΔSIAH1 transfected cells (*n* = 4), and β-catenin/Vinculin ratios in non-transfected (*n* = 7), SIAH1-transfected (*n* = 7), and ΔSIAH1 transfected cells (*n* = 5). Statistical significance was determined using the Wilcoxon signed-rank test (*P < 0.05, **P < 0.01, ***P < 0.001, ****P < 0.0001). **B** Mutational analysis of the AIRE degron. HEK-293 cells were transiently co-transfected with AIRE-MycHis (wild-type or the V123E and P125E mutants) and Flag-SIAH1 or Flag-ΔSIAH1. Cells were treated with or without the proteasome inhibitor MG-132 to evaluate the involvement of the ubiquitin-proteasome pathway. Relative AIRE/Vinculin densitometric ratios are shown for each condition

E3 ubiquitin ligases provide substrate specificity for polyubiquitination, often targeting proteins for proteasomal degradation. To investigate whether SIAH1 modulates AIRE stability, AIRE-expressing HEK-293 cells were transiently transfected with SIAH1. Western blot analysis, normalized to Vinculin, revealed a statistically significant reduction in AIRE levels upon SIAH1 overexpression (Fig. 8A). This decrease was highly reproducible across independent experiments. Notably, this effect was abrogated when cells were transfected with a catalytically inactive SIAH1 mutant (FLAG-∆SIAH1, lacking residues 1–89), demonstrating that the E3 ligase activity of SIAH1 is essential for AIRE degradation (Fig. 8A). A similar reduction was observed for β-catenin, a well-characterized SIAH1 substrate, further validating the assay.

To confirm the functional relevance of the identified degron (residues 119–125), degradation assays were repeated using the V123E and P125E mutants. As shown in Fig. 8B, these single-point mutations within the interaction motif impaired SIAH1-mediated degradation. Taken together, these data demonstrate that SIAH1 functions as an E3 ubiquitin ligase that targets AIRE for proteasomal degradation through a specific interaction with the motif spanning residues 119–125.

## Discussion

AIRE plays a pivotal role in the thymic medulla by ensuring an immunocompetent T-cell repertoire through the induction of promiscuous gene expression (PGE) in medullary thymic epithelial cells (mTECs) [7, 12–15, 17–19]. Mutations in the AIRE gene lead to the development of APS-1/APECED, a severe autoimmune disorder characterized by a breakdown in central tolerance [36]. Understanding the post-translational regulation of the AIRE protein is, therefore, essential for unraveling the mechanisms of immune tolerance and the development of autoimmunity. In this study, we identified and characterized the interaction between AIRE and members of the SIAH E3-ubiquitin ligase family, demonstrating that AIRE undergoes ubiquitination and subsequent proteasomal degradation mediated by SIAH1.

Our findings reveal a reciprocal regulation between AIRE and SIP (CACYBP). Stable AIRE-expressing HEK-293 clones showed consistent SIP upregulation, confirming that this process is AIRE-dependent. Conversely, SIP overexpression led to increased AIRE levels, suggesting a compensatory co-regulation mechanism potentially driven by their competition for SIAH binding.

The canonical SIAH-interacting motif (PxAxVxP) is present in SIP (residues 60–66; PAAVVAP) [37]. In human AIRE, we identified two putative motifs: AIRE_119-125_ (PKALVPP) and AIRE_416-422_ (PLLCVGP). Although the second motif contains a basic residue that has been suggested to enhance SIAH1 interaction [38], it features a leucine substitution for the canonical alanine at the second position. Our biochemical data and structural modeling demonstrate that AIRE interacts with SIAH exclusively through the first motif (residues 119–125). Truncated proteins lacking this region failed to co-immunoprecipitate with SIAH1/2, and AlphaFold models confirmed that AIRE_414-426_ cannot adopt the correct binding orientation. These results indicate that a canonical sequence is more critical for SIAH1 recruitment than the presence of flanking basic residues. Furthermore, competitive binding assays with purified proteins confirmed that AIRE and SIP directly compete for the same SIAH1 binding site.

The AIRE PKALVPP motif is in a structurally flexible region between the CARD and SAND domains, adjacent to the bipartite nuclear localization signal (NLS; residues 110–114 and 131–133). This motif is highly conserved across mammals (humans, mice, rats, and simians), with only minor variations in species such as horses, cows, and pigs. In contrast, the SIP motif remains entirely unchanged across mammalian evolution. While many proteins, such as ACK1 [39], OBF1 [40], DCC [41] and KLF10 [37], utilize this canonical motif for SIAH interaction, others like Axin-1 [42] or KIF22 [43] rely on non-canonical sequences. Our study highlights that AIRE belongs to the former group, utilizing a conserved, canonical degron to regulate its intracellular stability.

Our results demonstrate that AIRE undergoes SIAH1-mediated degradation. Although the reduction in protein levels was modest, it was remarkably consistent across both transient and stable transfection models. In contrast, SIAH2 did not show a consistent effect on AIRE stability; while some experiments suggested a decrease in AIRE abundance, others showed an increase, indicating that SIAH2 may not effectively degrade AIRE under the tested conditions. However, a potential degradative role for SIAH2 in different cellular contexts cannot be entirely excluded. To validate the specificity of these findings, we analyzed β-catenin, a well-known substrate of the SIP/SIAH1 pathway. The degradation kinetics observed for β-catenin mirrored those of AIRE, further supporting the role of SIAH1 as a regulator of AIRE stability. Notably, AIRE expression also correlated with reduced β-catenin levels, likely due to the AIRE-induced upregulation of SIP.

The interaction with SIAH1 appears to be functionally distinct from other AIRE-associated E3 ligases. For instance, FBXO3 co-localizes with AIRE in nuclear bodies to promote its transcriptional activity through ubiquitination [44]. In contrast, the AIRE–SIAH association is predominantly cytoplasmic, and we found no evidence of nuclear co-localization. Therefore, SIAH1 is unlikely to modulate AIRE’s transcriptional functions directly, acting instead as a regulator of its protein turnover and steady-state levels.

Furthermore, we cannot exclude the possibility that the interaction with SIAH proteins is required for AIRE to execute alternative functions. This could involve AIRE acting as a scaffolding component within a degradation complex, analogous to the role described for SIP. Alternatively, it was previously proposed that AIRE itself might possess E3-ubiquitin ligase activity [45], however, this hypothesis was largely dismissed following the NMR structural analysis of the PHD1 domain [46]. In addition, the AIRE–SIAH1 interaction may be linked to apoptosis induction. It is well established that AIRE⁺ mTECs frequently undergo apoptosis. Interestingly, SIAH1 is known to interact with S-nitrosylated GAPDH, facilitating its nuclear import to trigger cell death [47]. AIRE-induced apoptosis requires both the CARD domain and the nuclear translocation of GAPDH [48]. Our data show that the SIAH1-interacting motif (degron), in conjunction with the CARD domain, is essential for AIRE-mediated apoptosis. This suggests a model where SIAH1 regulates AIRE abundance in cells committed to the apoptotic pathway, potentially abrogating its transcriptional role once the cell has reached its final developmental stage.

SIAH1 and SIP are integral components of the β-catenin degradation pathway [49], and SIP has been shown to be essential for thymocyte development [50]. Recent studies have further demonstrated that SIAH1 mediates the degradation of Axin—the primary scaffolding protein of the β-catenin destruction complex—through a direct interaction [42, 51]. Notably, while the ubiquitination of β-catenin by SIAH requires the adaptor function of SIP, Axin ubiquitination is SIP-independent. Consequently, AIRE-induced upregulation of SIP may act as a rheostat for β-catenin levels. Given that AIRE directly interacts with SIAH1, the increase in intracellular SIP could represent a compensatory response to compete for SIAH1 binding. Thus, AIRE expression potentially modulates Wnt/β-catenin signaling by influencing both SIP abundance and its availability for SIAH1-mediated complexes.

This AIRE–SIAH1 axis may extend beyond the thymus, as both proteins are co-expressed during spermatogenesis and may play a role in meiotic regulation [5, 52]. Despite their high sequence homology, SIAH1 and SIAH2 exhibit distinct substrate specificities, which likely explains the inconsistent degradation of AIRE by SIAH2 observed in our assays. Furthermore, their biological roles are divergent: SIAH1 functions as a tumor suppressor [53], whereas SIAH2 acts as a proto-oncogene, driving progression in various malignancies, including melanoma, lung, breast, pancreatic, prostate, and liver cancers [54–60]. Therefore, the differential regulation of these ligases may significantly impact AIRE protein stability and its downstream physiological effects.

## Conclusion

In summary, our findings identify a novel regulatory pathway for AIRE mediated by SIAH1. We have demonstrated that AIRE undergoes SIAH1-dependent ubiquitination and proteasomal degradation through a specific, conserved SIAH-interacting motif (degron). This regulatory module not only advances our understanding of AIRE’s protein turnover and its role in apoptosis but also identifies the AIRE–SIAH1 interaction as a potential therapeutic target for APECED patients, particularly those carrying mutations that disrupt AIRE’s functional stability.

## Supplementary Information

Below is the link to the electronic supplementary material.

Supplementary Material 1

## Data Availability

Data will be made available on request.

## Abbreviations

AIRE: Autoimmune regulator
APS-1: Autoimmune polyglandular syndrome type 1
APECED: Autoimmune polyendocrinopathy candidiasis ectodermal dystrophy
FBS: Fetal bovine serum
mTECs: Medullary thymic epithelial cells
mDCs: Medullary dendritic cells
pMHC: Peptide-MHC complex
SIP: Siah-1-interacting protein
SIAH1: Seven-in-absentia homolog 1
SIAH2: Seven-in-absentia homolog 2
TRAs: Tissue-restricted antigen
