# Supplementary material for: The autoimmune regulator (AIRE) is a target of the E3-Ubiquitin ligase Seven-in-absentia homolog 1 (SIAH1)
Source: Apoptosis. 2026 Jul 14;31(8):193. doi: 10.1007/s10495-026-02398-9 (PMC13368847; doi:10.1007/s10495-026-02398-9)
Supplement: Supplementary file 1 — Supplementary Material 1 [file 10495_2026_2398_MOESM1_ESM.docx]

**Supplementary information**

**Figure S1.** CACYBP transcription is increased in AIRE^+^ stable transfected cells. PCR amplification from cDNA of *CACYBP* gene and *GAPDH* gene of HEK293 cells, HEK293-AIRE^+^ and HEK293-AIRE-MycHis^+^ clones. CACYBP/GAPDH ratios of the band densitometries are shown.

**Figure S2.** CACYCP/SIP increases the amount of intracellular AIRE. HEK-293 cells stably transfected with human AIRE gene were transiently transfected with the human SIP-FLAG. Figure shown the western blot analysis of AIRE expression after 48h of SIP-FLAG transfection. Bar diagram shows the AIRE/Vinculin ratio of the band densitometry in reference to AIRE-HEK-293 cells non- transfected with CACYBP/SIP.

**Figure S3.** AIRE protein sequence presents two possible SIAH binding motifs. The putative SIAH motifs are highlighted in red. First SIAH motif spans residues 119 to 125. Second SIAH motif spans residues 416 to 422.

**Figure S4.** SIP competes with AIRE to interact with SIAH1. Five μl of StII-ΔSIAH1 (SIAH1 lacking the first 89 residues) coupled to StII-G-Sepharose was incubated with 500 μl of SIP-6xHis purified protein for 2 h at RT or with PBS. Then, 500 μg of HEK293-AIRE cell lysates were added and incubated on rotation overnight at 4ºC. Samples were washed and analyzed by SDS-PAGE elecroforesis and Coomassie blue staining for SIP and SIAH1 and WB for AIRE. INPUT: Total cell lysate (1/50) from HEK293-AIRE^+^ alone or in combination with (1/500) of CACYBP/SIP-6xHis purified protein.

**Figure S5.** Predicted LDDT (pLDDT, values from 0 to 100) and matrix of Predicted Aligned Errors corresponding to the AlphaFold models. A) SIAH-SIP58-70, B) SIAH-AIRE117-129 and C) SIAH-AIRE414-426. Residue numbers from 1 to 158 in the plots correspond to SIAH, while those from 159 to 171 correspond to the peptide. The pLDDT predictor is a per-residue model confidence score, indicating the level of confidence in the residue's local structure. The predicted aligned errors indicate the model's confidence in the relative positions of pairs of residues, and thus in the interaction between SIAH and the peptide. More specifically, assuming residue y (vertical axis of the matrix) could be positionally aligned to the correct structure, the color in the plot represents the error (in Ångströms) that the model predicts for the position of residue x (horizontal axis). Low predicted errors are given a dark-green color (side bar). In the pLDDT plots the peptide is represented by the last peak, with the lower values flanking it corresponding to the unstructured tails of the peptide. The local structures of PAAVVAP (SIP60-66), PKALVPP (AIRE119-125) are predicted with high confidence (pLDDT > 90, pLDDT = 87 for AIRE's P125), while the structure of the entire AIRE414-426 is predicted with very low confidence (pLDDT < 50). The horizontal and vertical bands in the residue range 159-171 in the matrix of predicted aligned errors indicate clearly that while the confidence of the model on the interaction between SIAH and the peptide is high for SIP58-70 and AIRE117-129, it is very low for AIRE414-426.

**Figure S6.** Detail of the hydrogen bonding (dashed yellow lines) between SIAH and the SIP and AIRE117-129 peptides, illustrating the formation of a parallel β-sheet in both cases: A) crystallographic structure of SIAH-SIP59-67 (downloaded from the Protein Data Bank, https://www.rcsb.org/, entry 2A25); B) AlphaFold modeled structure of SIAH-SIP58-70; C) AlphaFold modeled structure of SIAH-AIRE117-129. Gold: SIAH residues in sticks representation; green: peptide residues. The representations include only the SIAH residues at less than 5 Ångströms from the binding residues of the peptide. SIAH residues Thr156 and Met180, which could be partly responsible for the non-binding of AIRE414-426, are indicated in panel A.

**Figure S7.** Structure of the complex between SIAH and the Leu418Ala mutant of AIRE414-426, as modeled by AlphaFold. A) Superposition of the crystallographic structure of the complex SIAH-SIP59-67 (downloaded from the Protein Data Bank, https://www.rcsb.org/, entry 2A25) with the modeled structure. Gold: crystallographic SIAH structure, the dashed line is indicative of an unresolved loop; green: crystallographic SIP59-67 structure; orange: modeled SIAH structure; blue: modeled AIRE414-426L418A structure. The panel shows both a ribbon representation, illustrating the β-strand secondary structures, and a sticks representation of the side chains. The representation includes SIAH residues 162-180, which constitute the binding surface, and 9 residues of each of the peptides (corresponding to the 9 residues resolved in the crystallographic structure; residues common to the two peptides are shown in bold): KPAAVVAPI (SIP59-67) and HPLACVGPE (AIRE415-423L418A). Predicted LDDT (pLDDT, values from 0 to 100) and matrix of Predicted Aligned Errors (in Ångströms) corresponding to the AlphaFold model (see Figure S1 for an explanation of these predictors).

**Figure S8.** Representative work flow of spontaneous and etoposide-induced apoptosis analysis induced by different AIRE constructs.

**Methodology figures S1 and S4**

**Figure S1**

About 5 × 10^6^ cells were used for RNA extraction, isolated using the RNeasy Micro Kit (Qiagen) with on-column DNase digestion using an RNase-free DNase set (Qiagen) following the manufacturer’s instructions. All purified RNA samples were processed into single-stranded cDNA using oligo(dT) primers and M-MLV reverse transcriptase. GAPDH and CACYBP/SIP PCR was performed using manually designed primers. Amplification was performed as follows: initial denaturation step at 95 ◦C for 2 min, twenty eight cycles of 95ºC for 30 s, 65ºC for 30 s, and 72ºC for 30 s for GAPDH PCR or twenty eight cycles of 95ºC for 30 s, 56ºC for 30 s, and 72ºC for 30 s for GAPDH PCR. The amplified products were visualized on a 1% TAE/agarose gel with MidoriGreen Stain 100X. Fiji (ImageJ) software was used for the densitometry analysis.

**Figure S4**

Competition assay. StII-ΔSIAH1 was initially captured with StII-G-Sepharose protein up to two hours on roation then the G-Sepharose was washed three times with 1mL of Isotonic Lysis Buffer (20mM Tris-HCl pH 8.0 150mM NaCl Protease Inhibitor Cocktail (C0mplete, Roche)) and were centrifuged 6000 xg, 2 min, 4ºC. About 5 µg of StII-ΔSIAH1 captured with StII-G-Sepharose protein was used in combination with 500 µg of CACYBP/SIP-6xHis purified protein, both proteins were incubated at least 2h on rotation. Another 5 µg of StII-ΔSIAH1 captured with StII-G-Sepharose protein was used alone as a control. Then about 500 µg of HEK293-AIRE cell lysates were added to both samples and were incubated O/N on rotation at 4ºC. Samples were washed 5 times with 400 µL of Isotonic Lysis Buffer and were centrifuged 6000 xg, 2 min, 4ºC. 40 µL of RBS5X were added and boiled to elute the proteins and then were analysed by electrofesis and western blot. 10% of the total cell lysate and the 1% of CACYBP/SIP-6xHis purified protein were used as INPUT samples.


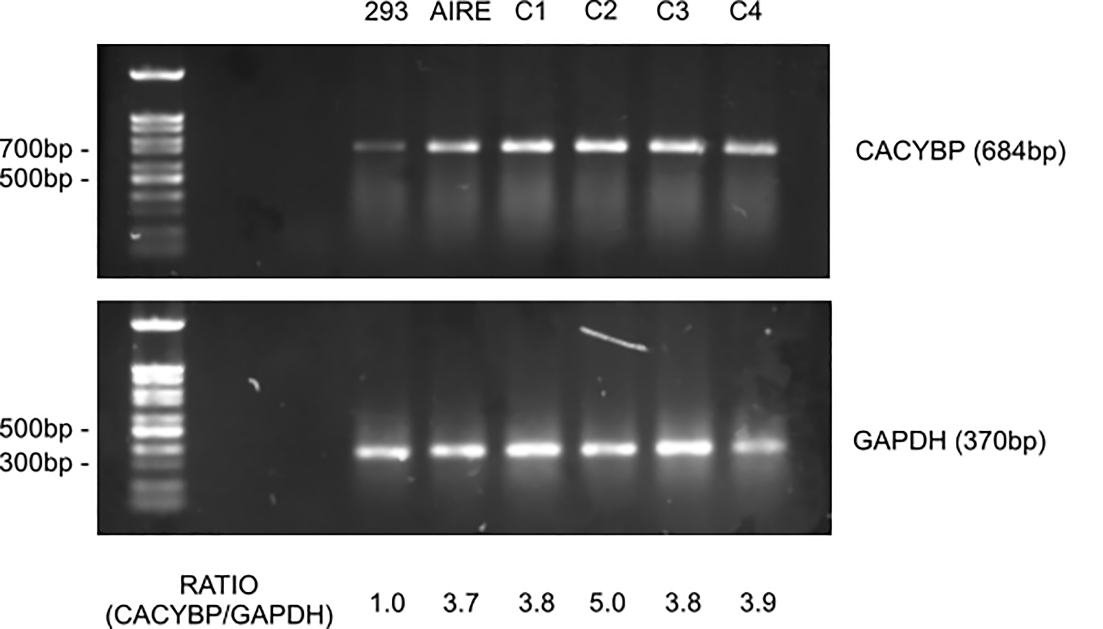


Figure S1


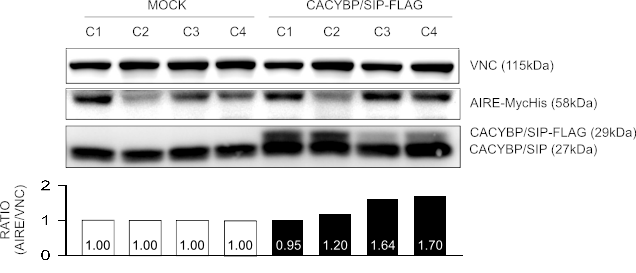


Figure S2


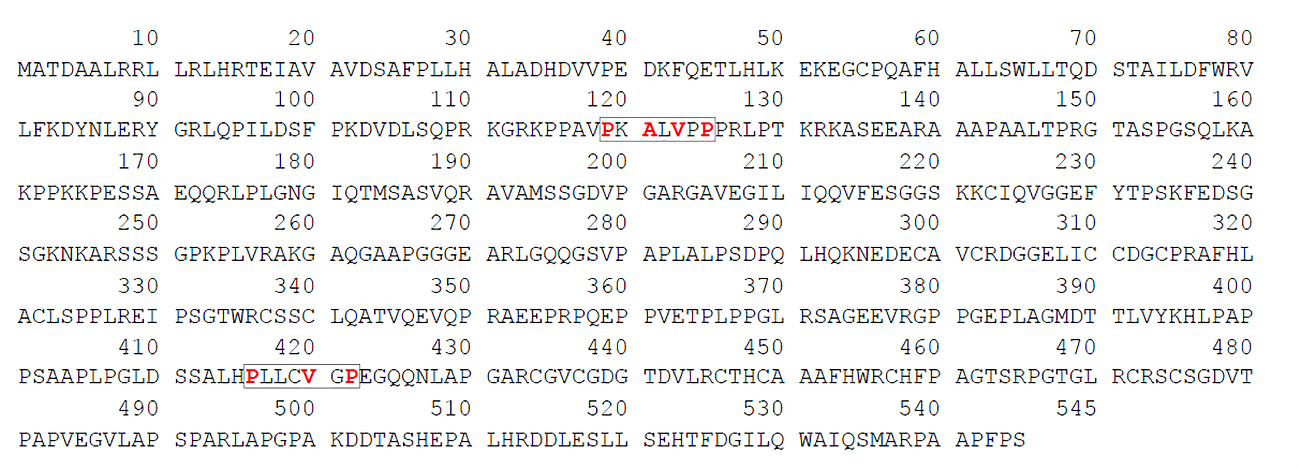


Figure S3


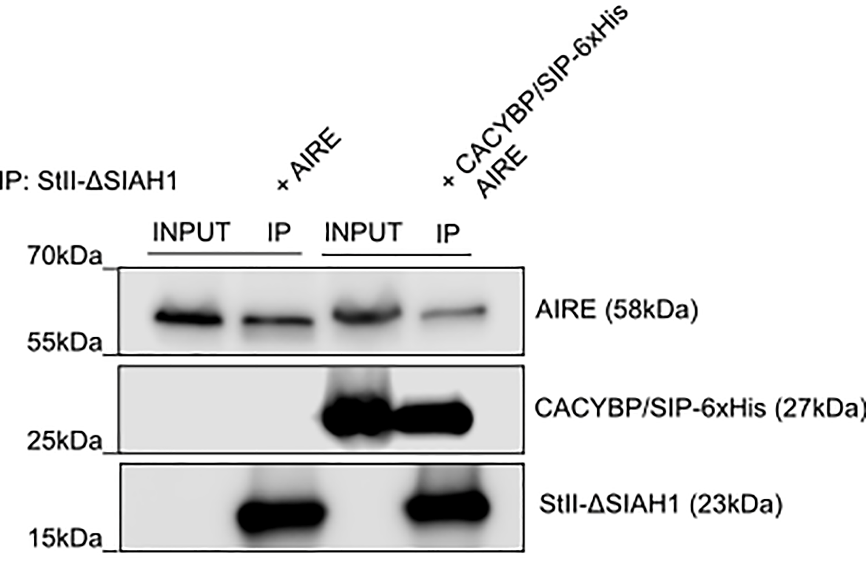


Figure S4


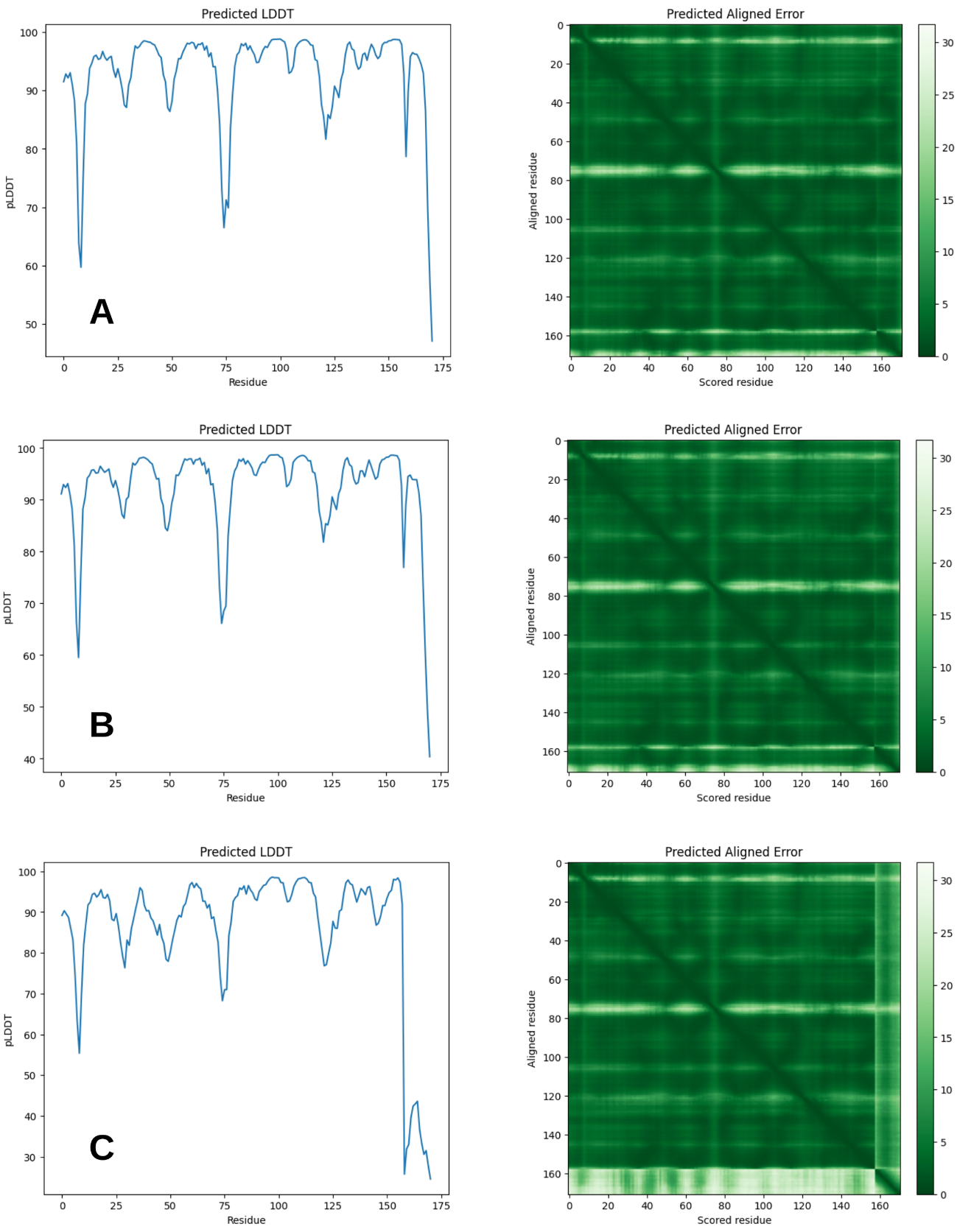


Figure S5


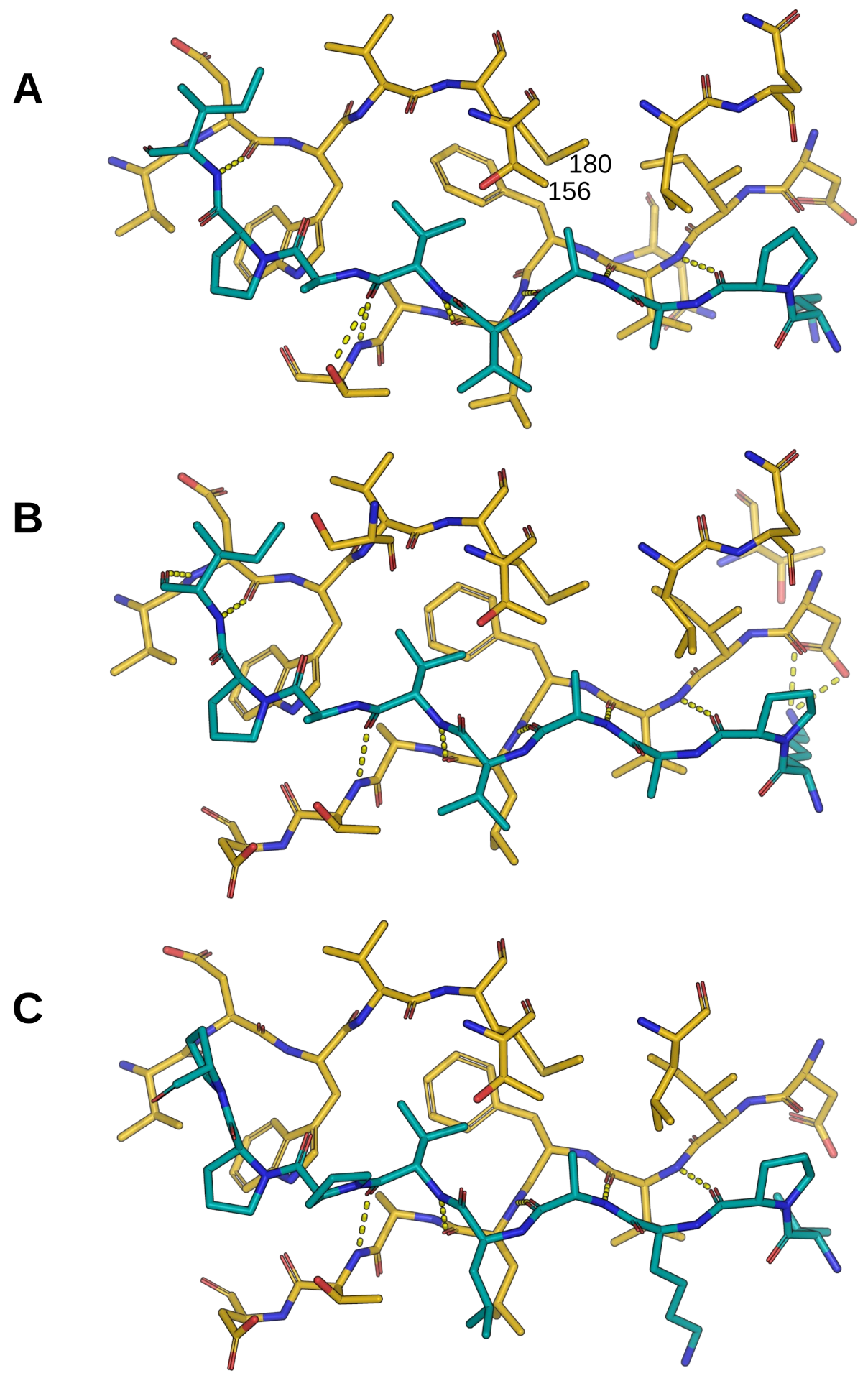


Figure S6


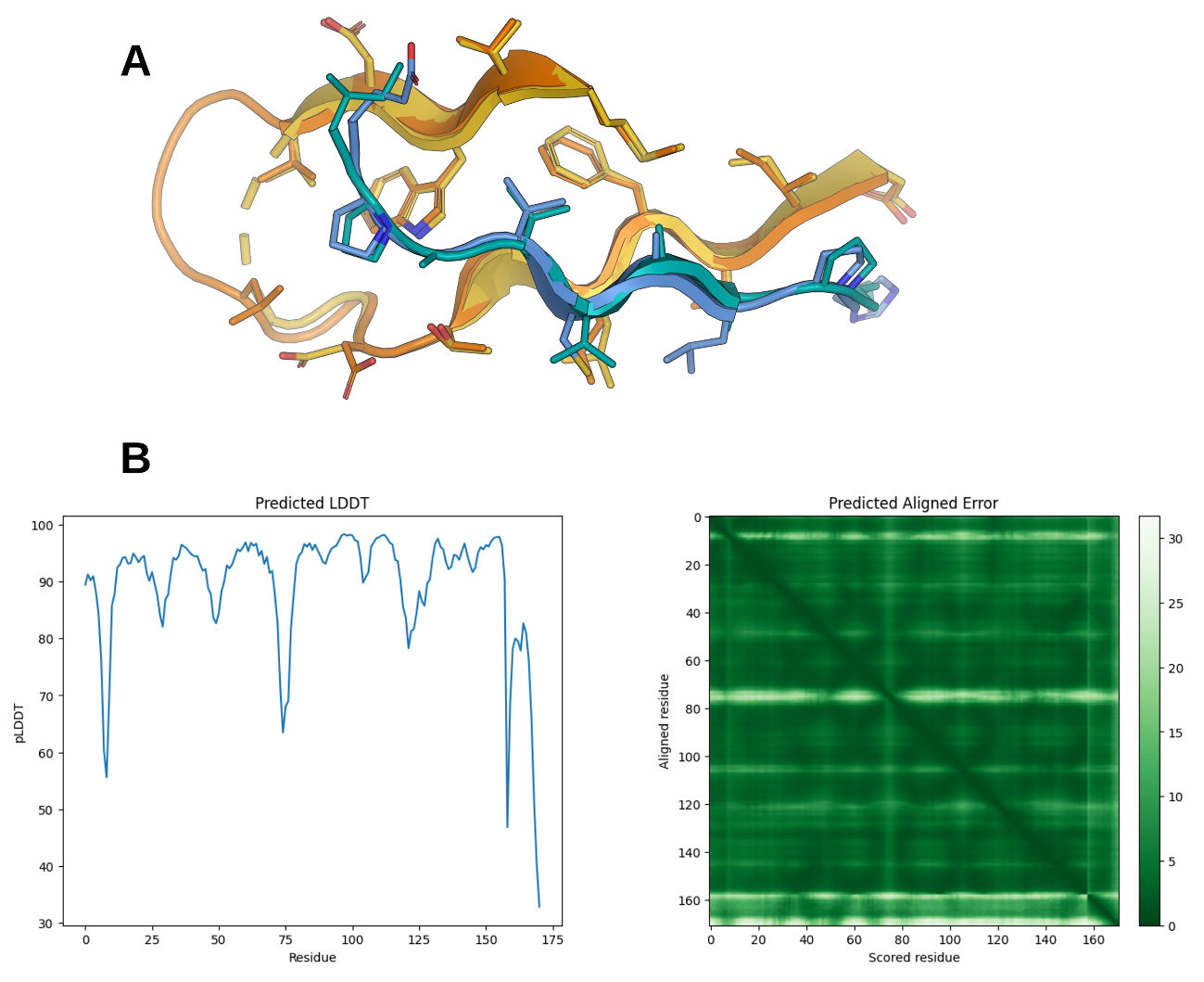


Figure S7


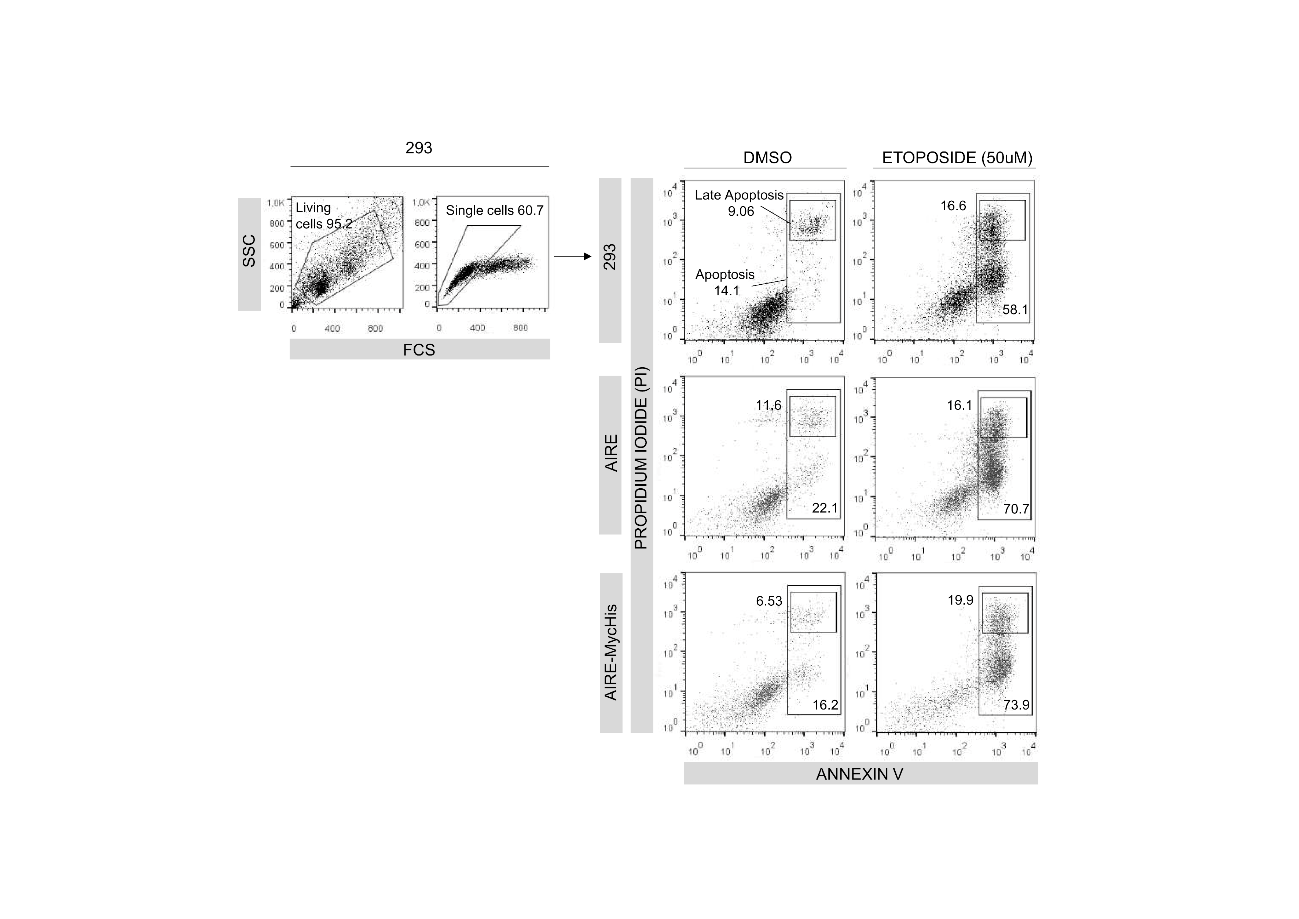


Figure S8
